# Corticosterone-mediated regulation and functions of miR-218-5p in rat brain

**DOI:** 10.1038/s41598-021-03863-y

**Published:** 2022-01-07

**Authors:** Yuta Yoshino, Bhaskar Roy, Yogesh Dwivedi

**Affiliations:** 1 Department of Psychiatry and Behavioral Neurobiology, University of Alabama at Birmingham, Birmingham, AL 35294 USA; 2 UAB Mood Disorder Program, Division of Behavioral Neurobiology, Department of Psychiatry and Behavioral Neurobiology, UAB Depression and Suicide Center, University of Alabama at Birmingham, SC711 Sparks Center, 1720 7th Avenue South, Birmingham, AL USA

**Keywords:** Diseases, Neuroscience, Epigenetics in the nervous system, Stress and resilience

## Abstract

Chronic stress is one of the key precipitating factors in major depressive disorder (MDD). Stress associated studies have underscored the mechanistic role of epigenetic master players like microRNAs (miRNAs) in depression pathophysiology at both preclinical and clinical levels. Previously, we had reported changes in miR-218-5p expression in response to corticosterone (CORT) induced chronic stress. MiR-218-5p was one of the most significantly induced miRNAs in the prefrontal cortex (PFC) of rats under chronic stress. In the present report, we have investigated how chronic CORT exposure mechanistically affected miR-218-5p expression in the rat brain and how miR-218 could trigger molecular changes on its downstream regulatory pathways. Elevated expression of miR-218-5p was found in the PFC of CORT-treated rats. A glucocorticoid receptor (GR) targeted Chromatin-Immunoprecipitation (ChIP) assay revealed high GR occupancy on the promoter region of Slit3 gene hosting miR-218-2 in its 3rd intron. RNA-sequencing data based on RNA Induced silencing Complex Immunoprecipitation (RISC-IP) with AGO2 in SH-SY5Y cells detected six consistent target genes of miR-218-5p (APOL4, DTWD1, BNIP1, METTL22, SNAPC1, and HDAC6). The expression of all five genes, except APOL4, was successfully validated with qPCR in CORT-treated rat PFC. Further, Hdac6-based ChIP-seq experiment helped in mapping major genomic loci enriched for intergenic regions in the PFC of CORT-treated rat. A proximity-based gene ontology (GO) analysis revealed a majority of the intergenic sites to be part of key genes implicated in central nervous system functions, notably synapse organization, neuron projection morphogenesis, and axonogenesis. Our results suggest that the upregulation of miR-218-5p in PFC of CORT-treated rats possibly resulted from GR biding in the promoter region of Slit3 gene. Interestingly, Hdac6 was one of the consistent target genes potentially found to regulate CNS related genes by chromatin modification. Collectively, these findings establish the role of miR-218-5p in chronic stress and the epigenetic function it plays to induce chromatin-based transcriptional changes of several CNS genes in triggering stress-induced disorders, including depression. This also opens up the scope to understand the role of miR-218-5p as a potential target for noncoding RNA therapeutics in clinical depression.

## Introduction

Chronic stress, both in childhood and in adult life, often leads to maladaptive changes in stress responsive pathways including an immediate elevation of sympathetic nervous tone and a delayed hypothalamic–pituitary–adrenal (HPA) axis activation^1^. Clinical evidence shows that increased vulnerability to mental disorders is significantly associated with prolonged stress^2^ and there is a strong relationship between chronic stress and the onset of MDD^3^. Chronic stress leads to repatterning brain circuitry function through a cascade of gene activation and deactivation. In response to stress, the release of adrenocorticotropic hormone (ACTH) subsequently activates the secretion of corticotropin-releasing factor (CRF), which induces glucocorticoid synthesis and their systemic release (cortisol in humans and corticosterone [CORT] in rodents)^4^. When glucocorticoid binds to its cognate nuclear receptor, the glucocorticoid receptor [GR]), it triggers a series of downstream events, most notably the initiation of gene transcription in the limbic and pre-limbic areas of the brain^5,6^. Of several brain areas, the prefrontal cortex (PFC) is strongly involved in cognitive and executive functions and participates in the regulation of adaptive stress responses. Pathological changes to these adaptive responses in PFC have often been studied as the cause of depressogenic activity through the disruption of HPA axis^7–9^. In rodents, chronic stress causes structural atrophy in dendritic projects of neurons and a significant synaptic loss or repatterning in PFC. Recently, large-scale transcriptomic studies in rodents have pointed out the consequential role of chronic stress on cellular apoptosis, proliferation, cell cycle control, axonal guidance and cell migration in response to GR-induced gene expression changes. Interestingly, it has been shown that many of the target genes and their upstream or downstream regulators in the PFC could potentially be linked to the overall atrophy/remodeling of neuronal structure and functions. It is important to note that these molecular and cellular changes are strongly reflected in behavioral manifestations associated with depressive phenotype^10^.

Recently, non-coding RNAs, particularly miRNAs, have been studied extensively, not only for their regulatory roles in neural plasticity and higher brain functioning, but also in the context of disease pathophysiology. miRNAs are defined as short, non-protein-coding sequences between 21 and 25 bp that regulate gene expression through RNA silencing^11^. One strand of miRNA/miRNA* duplex loads into Argonaute homologue protein (Ago) to make RNA-induced silencing complex (RISC), and could regulate the gene expression either through mRNA translation or transcript degradation^12,13^. The most common location on genes for miRNA to bind is the 3' untranslated region (UTR); however, studies indicate that 5'UTR, as well as coding regions within the gene, can both contain miRNA binding sites^14^. Genome-wide analysis of miRNA binding sites has revealed that miRNAs can regulate more than half the entire genome^15^. These miRNA targets are often highly enriched in disease phenotypes that are at high risk for chromosomal or genetic variations. Since miRNAs act in concordance with other regulatory molecules, they collectively orchestrate a complex molecular circuit that is especially important in complex diseases, like mental disorders, where polygenic influences are mostly common^16^.

In a series of studies, we have shown that the expression of miRNAs is significantly altered in various brain areas of MDD subjects. We also found that miRNAs are involved in stress resiliency and vulnerability to develop depressive phenotypes in rats. More recently, we showed that a select group of miRNAs were significantly altered in the PFC of rats who were given CORT chronically and showed depression-like behavior^17^. Of those miRNAs, we reported that miR-124-3p regulated stress-related genes, such as Nr3c1, with in vivo and in vitro interaction experiments^18^. One of the top upregulated miRNAs in this study was miR-218-5p, which is processed from miR-218-2 transcript and housed within the intronic region of Slit3 coding gene^19^. It has been shown earlier that the other member of Slit gene family, i.e., Slit2 harbors the intronic miR-218-1 which encodes the other isoform of miR-218 family (miR-218-1-3p)^20^. Both these isoforms are rooted to Slit-Robo signaling axis with functional role in depression-/anxiety-like behaviors as previously reported in adult mice model^21^. In-silico analysis revealed that miR-218-5p shared multiple stress responsive target genes with miR-124-3p^17^. However, how CORT modifies the expression of miR-218-5p and how miR-218-5p regulates stress-related genes at the downstream level, are not understood. In the present study, we mechanistically examined the regulation of miR-218-3p gene by CORT; the functional impact of altered expression of miR-218-3p on target genes; and the role of specific target genes in stress responsiveness. For this, we investigated: (1) CORT-mediated effects on the promoter regions of miR-218 gene; (2) consistent target genes of miR-218-5p using overexpression analysis in-vitro; (3) validation of miR-218-5p target genes in PFC of CORT treated rats; and, (4) impact of Hdac6, one of the consistent target genes, on downstream genes through chromatin modification.

## Methods

### Animals

All the experiments were carried out in accordance with the National Institutes of Health guide for the care and use of laboratory animals. The Animal Care Committee of the University of Alabama at Birmingham approved this study. The study is reported in accordance with ARRIVE guidelines (https://arriveguidelines.org).

Virus-free Sprague–Dawley male rats, initially weighing 220–250 g, were used. Rats were housed in groups of three under standard laboratory conditions (temperature 21 ± 1 °C, humidity 55 ± 5%, 12-h light/dark cycle). Animals were provided free access to food. Rats were acclimatized for 1 week before the start of experiments. Six animals in each group were used for miRNA expression analysis, 5–6 animals in each group were used for chromatin immunoprecipitation (ChIP) based qPCR experiments and target gene expression profiling were done in 13–22 animal in each group.

### Corticosterone treatment

The procedure for corticosterone treatment has been described in our earlier publication^22^. Rats, under light halothane anesthesia, were subcutaneously implanted with corticosterone pellets containing 100 mg of corticosterone in a cholesterol base (Innovative Research of America, Sarasota, FL, USA). These corticosterone pellets can maintain a physiological serum concentration of corticosterone for 21 days. The release of CORT after implantation of a 100-mg corticosterone pellet is 4.76 mg per day. Control rats underwent an identical surgery procedure with implantation of a cholesterol base pellet. Rats were decapitated 21 days after pellet implantation. This procedure has been published in our earlier study^22^.

The trunk blood was collected on ice and centrifuged; then, the serum was stored at − 80 °C until the assays were performed. Serum corticosterone levels were measured by a commercially available radioimmunoassay kit (ICN Biomedical, Inc., Cleveland, OH, USA). Brains were removed quickly after the blood was taken. Prefrontal cortex (PFC) was dissected out and immediately stored at − 80 °C until analyzed. Rats were decapitated between 09:00 h and 11:00 h, corresponding to 3–5 h after lights on. All experiments were performed in 6–12 rats per group.

### Cell culture and transfection of miR-218-5p mimic oligo

SH-SY5Y cells (Human neuroblastoma cells; ATCC CRL-2266) were cultured in Dulbecco's Modified Eagle Medium (DMEM) containing 10% fetal bovine serum, 2 mM glutamine, and penicillin and streptomycin (10000U/ml). The cells were incubated at 37 °C in a 5% CO_2_ atmosphere and the medium were refreshed every 24 h. Transient transfection of miRNA mimic (Dharmacon, Lafayette, CO, USA) was performed using Lipofectamine RNAiMAX (Invitrogen, Grandsland, NY, USA) according to the manufacturer’s protocol. Double-stranded RNA oligos [rno-miR-218a-5p of mimic (C-320416-03), Dharmacon GE Life Sciences, Lafayette] or vehicle (as non-transfection control) was transfected into SH-SY5Y cells and harvested 48 h post-transfection. Lysates were stored in TRIzol for RNA isolating (as input) samples or in RIP (RNA Immunoprecipitation) lysis buffer (150 mM NaCl, 50 mM Tris–Cl pH 7.4, 1 mM EDTA, 1 mM DTT, 0.5% NP-40, 1 × Halt Protease Inhibitor Cocktail, 200 U/ml RNaseOUT) for conducting RNA-immunoprecipitation assay. It must be noted that rno-miR-218a-5p of mimic is for rat; however, it is also used in human cell line because the miR-218-5p sequence is exactly the same between human and rat (UUGUGCUUGAUCUAACCAUGU; http://www.mirbase.org/).

### RNA-immunoprecipitation assay (RNP-IP)

Cell lysates collected in RIP lysis buffer were used for RNA-immunoprecipitation assay following a protocol by^18,23^. Briefly, prewashed protein A/G beads (Santa Cruz Biotechnology, Dallas, TX, USA) were conjugated with Anti-Ago2 monoclonal antibody (MABE253, Millipore, Billerica, MA, USA) and were added with cell lysate. Following overnight incubation, the immunoprecipitated RNA-induced silencing complex (RISC) was used to extract overexpressed miR-218-5p and its bound targets mRNAs using TRIzol reagent as RNA-IP samples.

### RNA isolation

Total RNA from cell lysates was isolated with TRIzol reagent (Invitrogen, Carlsbad, CA, USA) according to the manufacturer's protocol with a few modifications to maximize yield of small RNAs. Glycogen (20 μg) was added to the RNA precipitation step which was allowed to proceed overnight at − 20 °C. RNA samples were quantified on a NanoDrop ND-1000 instrument and their purity was checked (260/280 nm; cutoff ≥ 1.8). Agarose electrophoresis was used to check the integrality of RNA samples.

### RNA sequencing and analysis

1–2 µg total RNA from each sample was taken for RNA-seq library preparation following the method described previoulsy^24^. Briefly, mRNA was isolated from total RNA with NEBNext Poly(A) mRNA Magnetic Isolation Module. rRNA was removed from the total RNA with a RiboZero Magnetic Gold Kit; the resulting pool was enriched for mRNA and subsequently used in RNA-seq library preparation using KAPA Stranded RNA-Seq Library Prep Kit (Illumina, San Diego, CA, USA). The library preparation procedure included: (1) fragmentation of the RNA molecules; (2) reverse transcription to synthesize first strand cDNA; (3) second strand cDNA synthesis incorporating dUTP; (4) end-repair and A-tailing of the double stranded cDNA; (5) Illumina compatible adapter ligation; and, (6) PCR amplification and purification for the final RNA-seq library. The completed libraries were qualified on Agilent 2100 Bioanalyzer for concentration, fragment size distribution (400–600 bp), and adapter dimer contamination. The amount was determined by absolute quantification qPCR method. The barcoded libraries were mixed in equal amounts and used for sequencing. The DNA fragments in well mixed libraries were denatured with 0.1 M NaOH to generate single-stranded DNA molecules, loaded onto channels of the flow cell at 8 pM concentration, and amplified in-situ using TruSeq SR Cluster Kit v3-cBot-HS (#GD-401–3001, Illumina). Sequencing was carried out using the Illumina HiSeq 4000 according to the manufacturer’s instructions. Sequencing was carried out by running 150 cycles.

Raw data files in FASTQ format were generated from the Illumina sequencer. To examine the sequencing quality, the quality score plot of each sample was plotted. Sequence quality was examined using the FastQC software. After quality control, the fragments were 5′, 3′-adaptor trimmed and filtered ≤ 20 bp reads with Cutadapt software. The trimmed reads were aligned to reference genome with Hisat 2 software. Based on alignment statistical analysis (mapping ratio, rRNA/mtRNA content, fragment sequence bias), it was determined whether the results could be used for subsequent data analysis. The expression levels (FPKM value) of known genes and transcripts were calculated using ballgown through the transcript abundances estimated with StringTie. The number of identified genes and transcripts per group was calculated based on the mean of FPKM in group (≥ 0.5). Differentially expressed gene and transcript analyses were performed with R package ballgown. The volcano plot was created based on FPKM values with web-based tools (https://paolo.shinyapps.io/ShinyVolcanoPlot/). The heat map was also created based on FPKM value by ClustVis^25^.

### Identification of consistent target genes of miR-218-5p based on input RNA-seq and IP RNA-seq results

The prominent target genes of miR-218-5p were determined by the following criteria: (1) significantly downregulated genes in input RNA-seq, and (2) significantly upregulated genes in RISC-IPed RNA-seq.

### ChIP-sequencing (ChIP-Seq) and ChIP-qPCR (qChIP)

Briefly, 30 mg of frozen PFC tissue was cut into small pieces. The 1% formaldehyde in ice-cold phosphate buffered saline (PBS) containing proteasomal inhibitors (1X complete protease inhibitor, 1 mM of PMSF [phenylmethylsulfonyl fluoride] and 25 μm MG-132) was added to brain pieces. The brain pieces were incubated for 10 min at 37 °C for cross-linking reaction. The cross-linking reaction was stopped by adding 125 mM glycine. Crosslinked tissue pieces were washed twice with ice-cold 1XPBS and suspended with SDS lysis buffer (1% SDS, 10 mM EDTA, and 50 mM Tris–HCl [pH 8.1]) containing protease and proteasome inhibitors for 10 min on ice bath. Brain lysates were sonicated using Active Motif Q800R sonicator (Amplitude: 25%, Pulse Duration: 30 s, pulse on time: 10 min, number of pulses: 20). The resulting homogenates were centrifuged for 10 min at 13,000×*g* at 4 °C to remove debris. The supernatants were diluted in ChIP dilution buffer with protease and proteasome inhibitors (0.01% SDS, 1.1% Triton X-100, 1.2 mM EDTA, and 16.7 mM Tris–HCl [pH 8.1]). Ten percent of the volume was stored at − 80 °C as ChIP-input sample. The rest of the samples were used for immunoprecipitation and were incubated overnight with 5 μg of antibody for HDAC6 (Cell Signaling [D21B10], Danvers, MA, USA) or glucocorticoid receptor (ThermoScientific, Waltham, MA, USA [MA1-1850]) with overhead rotation. After immunoprecipitation, 20 μl of the pre-washed Protein A magnetic beads (#73778, Cell Signaling, Danvers, MA, USA) were added to the samples, and chromatin-antibody complex was collected for 2 h at 4 °C with overhead rotation. Immuno-enriched chromatin-bead complex was sequentially washed with low salt buffer (0.1% SDS, 1% Triton X-100, 2 mM EDTA, 20 mM Tris–HCl [pH 8.1], and 150 mM NaCl), high salt buffer (0.1% SDS, 1% Triton X-100, 2 mM EDTA, 20 mM Tris–HCl [pH 8.1], and 500 mM NaCl), LiCl buffer (0.25 M LiCl, 1% NP-40, 1% deoxycholate, 1 mM EDTA, and 10 mM Tris–Hcl [pH 8.1]), and lastly Tris–EDTA (TE) buffer. Crosslinks were reversed by the addition of 200 mM NaCl and heating at 65 °C for 4 h. Similar steps were followed for storing ChIP input samples. Both the ChIP-IP and ChIP-input fraction DNAs were purified using phenol: chloroform: isoamyl alcohol method.

ChIP with HDAC6 antibody and corresponding input DNA samples were subjected to ChIP-seq. ChIP-seq libraries were sequenced with the Illumina HiSeq2000 sequencer (50-nucleotide pair-ended read) at the Heflin Center Genomics Core, University of Alabama at Birmingham. On the other hand, immunoprecipitated ChIP DNA with GR antibody and corresponding input DNA samples were subjected to relative quantification with EvaGreen dye-based chemistry. The control qPCR experiment was done using DNA from IgG antibody-based ChIP-IP and corresponding ChIP-input samples. All the primers are shown in Table S1. The input normalized fold enrichment was calculated according to the method described earlier^26^.

### Functional annotation of nearest gene from peak change in ChIP-seq

The nearest genes from the significantly altered peaks in ChIP-seq in each sample were used in standalone Cytoscape program to perform gene ontology (GO) analysis using ClueGO plugin. Display pathways were selected with p ≤ 0.05. Clustering was done based on the common functionality of genes enriched for specific term. The kappa score was set at 0.5 to define the term-term relationship (i.e., edges between the nodes).

### qPCR based mRNA and miRNA-specific gene expression

mRNA-specific cDNA was synthesized with the Oligo (dT)_18_ priming method. A mixture of 0.5 µg of total RNA, oligo (dT)_18_, dNTP, and double distilled water was incubated at 65 °C for 5 min and quickly chilled on the ice. Subsequently, the reaction mixture was added with 1× 1st strand synthesis buffer, 0.01 mM DTT, 2 U RNaseOut and 200 U M-MLV reverse transcriptase (Invitrogen, Grand Island, NY, USA) and incubated at 37 °C for 50 min. Finally, the reaction was inactivated at 70 °C. miRNA-specific cDNA synthesis was performed using poly(A)-tailing method and priming with oligo dT adaptor primer. qPCR-based relative expression was measured with EvaGreen chemistry (Applied Biological Materials, Canada). All the primer sequences are shown in Table S1. Gapdh was used as a normalizer for mRNA expression, whereas U6 was used for miRNA expression. Fold expression changes were calculated by Livak’s ΔΔCt method^27^.

### qPCR based miR-218 expression assay using in vitro cellular model with GR gain of function mutation

We used HEK-293 cell line to transfect GR overexpression clone (SinoBiologicals Inc., Wayne, PA, USA). In parallel, control group was prepared by transfecting the cell line with scramble plasmid. Forty-eight hours post-transfection, cells were harvested, and RNA was extracted as mentioned above. The extracted RNA was used in miRNA-specific cDNA preparation and subsequently used in qPCR with primers to determine miR-218 expression. The expression was normalized by U6 gene as reference. The fold-change was determined as mentioned above.

### Statistical analysis

The SPSS 25.0 software (IBM, USA) was used for statistical analyses. Shapiro–Wilk test was used to assess the normality of the data. The average differences of mRNA and miRNA expressions were assessed by the student’s t test. The difference of fold enrichment in ChIP experiment was also assessed by the student’s t test. Statistical significance was set at the 95% level (p ≤ 0.05).

## Results

### Serum corticosterone levels

Serum corticosterone levels were measured in sham and corticosterone-treated rats. The serum corticosterone levels in these rats were as follows: sham, 40.3 ± 0.10. 3; CORT, 109.8 ± 27.1 ng/ml, which was significantly higher in the CORT-treated group. As with our earlier studies^22,28^, corticosterone level was significantly higher (p < 0.001) in corticosterone pellet-implanted rats.

### miR-218-5p expression in CORT-treated rats

**Figure 1:**
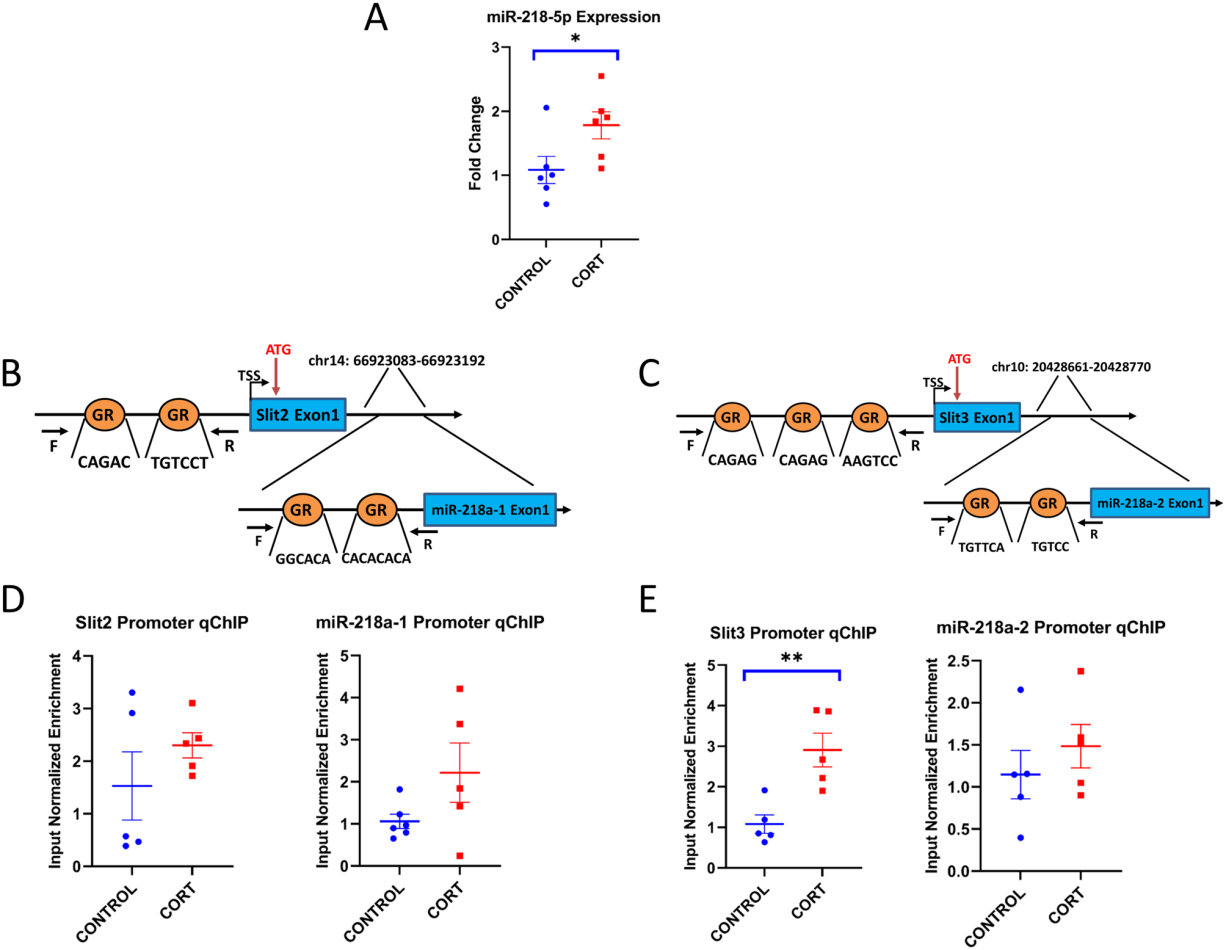
In vivo expression and promoter ChIP assay for miR-218 gene. (**A)** miR-218a-5p expression was significantly higher in the CORT-treated group compared with the vehicle control group. Values denote mean ± SEM. U6 was used as normalizer. *p < 0.05. (**B**,**C**) Schematic diagram of miR-218a-1 and -2 locus and ChIP assay for GR binding on those promoter regions. miR-218a-3101 and -2 genes are located in the intronic regions of Slit2 and Slit3 respectively. We selected promoter regions (up to1k bp from TSS) which included predicted GR biding sites by in-silico for qPCR experiments. (**D**) There are increasing trends of GR biding in the CORT-treated group of Slit2, miR-218a-1 and miR-218a-2. (**E**) Significant enrichment of GR was found in the CORT-treated group of Slit3 promoter. *p < 0.05 and **p < 0.005. F, forward; R, reverse; GR, glucocorticoid receptor.

To check miR-218-5p expression in CORT-treated rats, qPCR was performed in the PFC. Expression of U6 was not significantly different between control and CORT-treated groups. The miR-218a-5p expression was significantly elevated in the CORT-treated group (*p* = 0.031, Fig. 1A).

### In-vivo binding of glucocorticoid receptor (GR) on promoter regions of miR-218a-1, -2, Slit2, Slit3

To assess whether CORT affects miR-218-5p expression through GR, ChIP assay was performed in the promoter regions, including GR biding sites, predicted by Patch program v.1.0 (http://gene-regulation.com/pub/programs.html#patch). There are two forms of miR-218 coding gene: miR-218a-1 and miR-218a-2, located in intron regions of Slit2 and Slit3, respectively (Fig. 1B,C). The qPCR results showed the trends of enriched GR biding on Slit2 promoter in the CORT-treated group (*p* = 0.06), for miR-218a-1 (*p* = 0.19), but they did not reach significant levels (Fig. 1D). On the other hand, a significant enrichment of GR binding was found in CORT-treated group of Slit3 promoter (*p* = 0.001). However, for miR-218a-2 (*p* = 0.16) the expression increased, but it was not significant in the CORT treated group (Fig. 1E).

### Transfection of miR-218-5p mimic oligos

**Figure 2:**
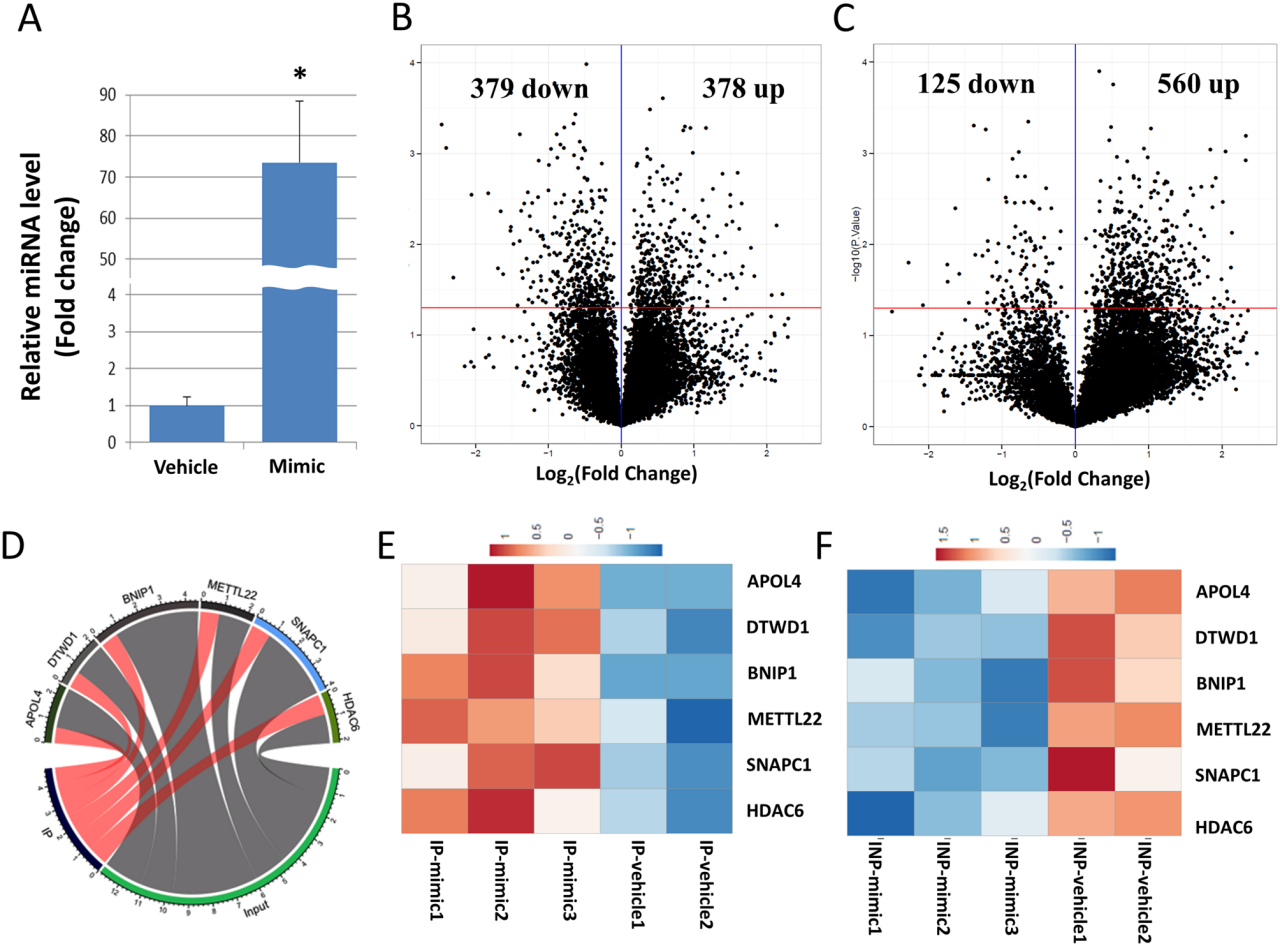
In vitro expression assay for miR-218-5p and RISC mediated target gene pulldown. (**A**) Relative expression profile of miR-218-5p in miR oligo transfected SH-SY5Y cells. Relative fold change analysis showed significantly higher expression of miR-218-5p in the mimic group compared to the vehicle group (t = 1.447, df = 3, p = 0.244). Values denote mean ± SEM. U6 was used as normalizer. *p < 0.005. (**B**) Volcano plot showing differential enrichment (up) and depletion (down) of target genes in RISC pull down (IP) complex. (**C**) Volcano plot showing differential expression profile of genes affected by miR-218-5p oligo overexpression based on input-RNA-seq results. (**D**) Six genes were detected as consistent target genes regulated by miR-218-5p overexpression. Consistent target genes were predicted by the following criteria: (1) significantly downregulated genes in the input RNA-seq and (2) significantly upregulated genes in the IP RNA-seq. In the chord diagram, the degree of interactions between input and IP groups on the six targets are shown with respective fold change values. The expression heat maps were created based on six genes with IP RNA-seq (**E**) and input RNA-seq (**F**).

SH-SY5Y cells were collected from the vehicle (n = 2) and mimic groups (n = 3), and qPCR was performed for validation of miR-218-5p overexpression. The relative miR-218-5p expression level was significantly higher in the miR-218-5p transfected group compared to the vehicle group (*p* = 0.033, Fig. 2A). The level of U6 was not significantly different between vehicle and mimic groups (*p* = 0.244).

### Input samples-based RNA-seq of coding transcripts and core analysis of downregulated genes

A total of 12,222 genes were detected in RNA-seq for input samples. The volcano plot was created using all the differentially regulated genes based on input RNA-seq and Ago-RISC IP-RNA-seq results (Fig. 2B,C). Based on input RNA-seq results (Fig. 2B), 378 genes were found significantly upregulated and 379 were significantly downregulated in the mimic group compared to the vehicle group (downregulated genes are shown in Table S2). The 379 downregulated genes were further used for subsequent analysis since miRNAs generally regulate mRNA expression inversely. In Fig. 2C, volcano plot showing differential enrichment (up) and depletion (down) of target genes in RISC pull down (IP) complex, 560 genes were found up and 125 genes were down in IP group.

### RNA-seq for IP samples to identify consistent target genes based on core analysis with the input RNA-seq and IP RNA-seq data

A total of 12,522 genes were detected in RNA-seq for IP samples. The volcano plot was created using all the genes (Fig. 2C). Out of 12,522 genes, 560 were significantly upregulated and 125 were significantly downregulated in the mimic group compared to the vehicle group (all upregulated genes are shown in Table S3). We focused on 560 upregulated genes for subsequent analysis because those genes should be packed in RISC complex under the overexpressed miR-218-5p condition and will be subsequently downregulated. The result of the putative target genes, that were downregulated in input RNA-seq and upregulated in IP RNA-seq experiments, was used to determine an overlapped set of genes. The overlapped six genes (APOL4, DTWD1, BNIP1, METTL22, SNAPC1, and HDAC6) were predicted to be experimentally consistent target genes. In the chord diagram, the degree of interactions between input and IP groups on the six targets are shown with respective fold-change values (Fig. 2D). Two expression heat maps (Fig. 2E,F) were created based on six genes as they were originally identified in IP- and input-RNA-seq from miR-218 transfected human neuroblastoma cell line (SHSY-5Y). Subsequently, five genes, except APOL4 were tested in the qPCR experiments in rat PFC because APOL4 gene doesn’t exist in rats (https://www.ncbi.nlm.nih.gov/gene/).

### Validation of consistent target genes in CORT-treated rats based on qPCR

**Figure 3:**
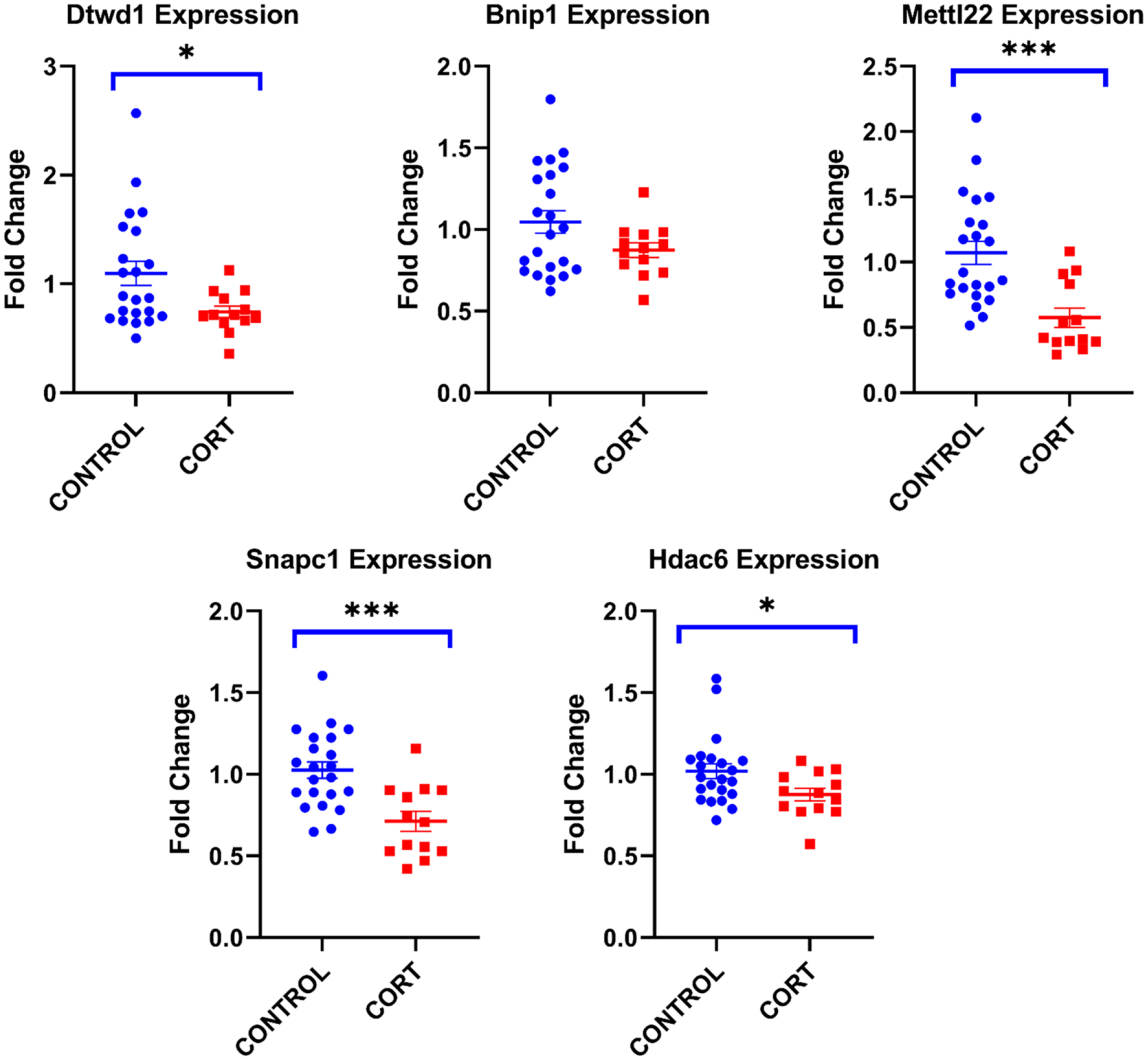
Fold change of consistent target genes in the PFC of CORT-treated rats. Target gene expression differences between control and CORT treated rats were measured following relative fold change method. All five genes (Dtwd1, *p* = 0.019; Mettl22, *p* < 0.005; Snapc1, *p* < 0.001; Hdac6, *p* = 0.03) showed significant expression down regulation in CORT rat PFC except Bnip1 (*p* = 0.12) Values denote mean ± SEM. Gapdh expression values were used as normalizer. *p < 0.05 and **p < 0.005.

The qPCR of five consistent target genes was done to investigate whether these genes are altered in PFC of CORT-treated rats. The level of Gapdh (control vs CORT = 22 vs 13, *t* = -2.005, *df* = 33, *p* = 0.053) was not significantly different between control and CORT-treated groups. As shown in Fig. 3, all 4 consistent target genes were significantly downregulated in the CORT-treated group (Dtdw1, *p* = 0.019; Mettl22, *p* < 0.005; Snapc1, *p* < 0.001; Hdac6, *p* = 0.03) except Bnip1 (*p* = 0.12).

### Mapping the genomic region bound by Hdac6 revealed by ChIP-seq

**Figure 4:**
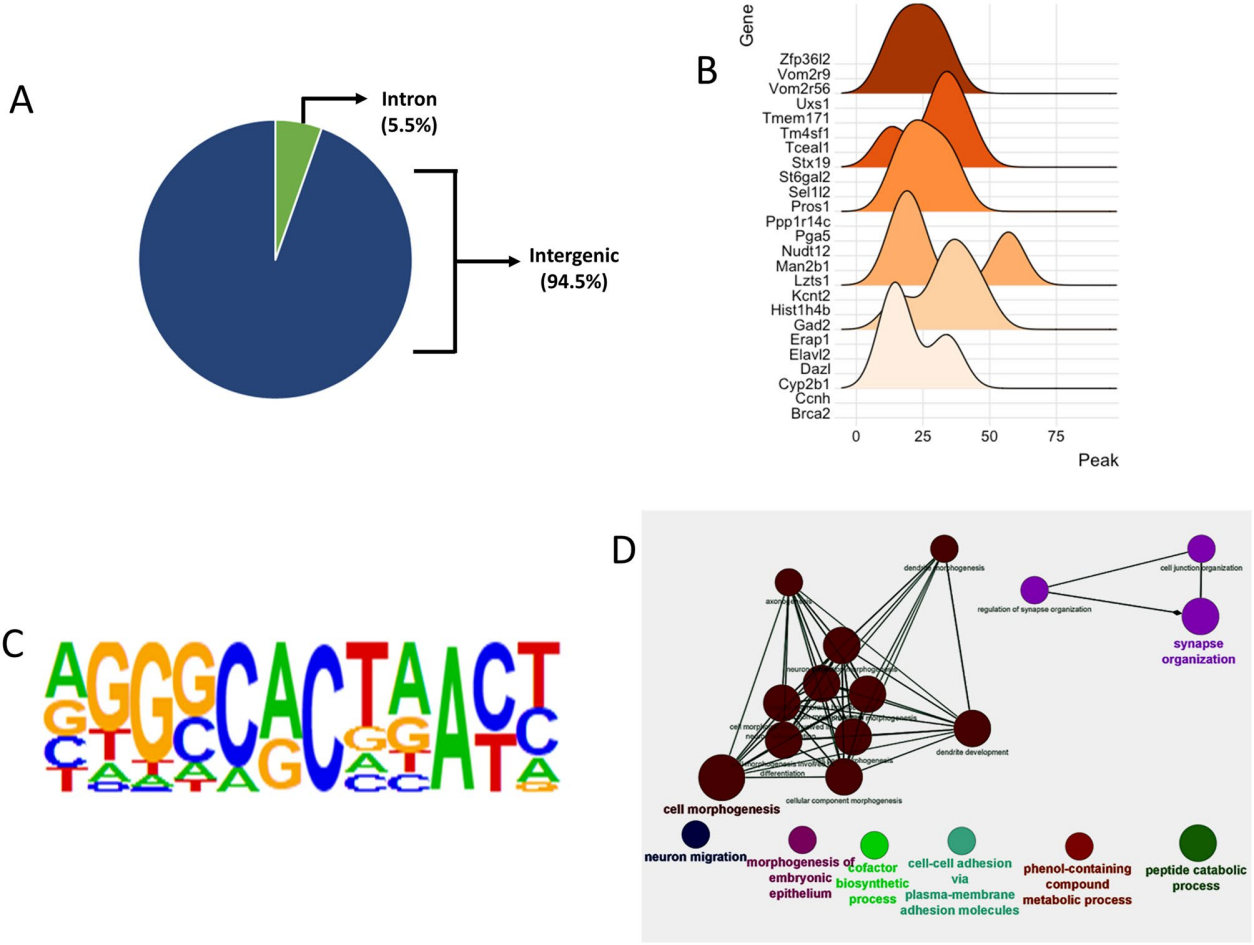
HDAC6-based ChIP-seq results in the PFC of CORT-treated rats. (**A**) Proportion of peak change regions in CORT treated rat PFC. (**B**) Ridgeline plots show the HDAC6-based chromatin peaks at the genomic scale. Chromatin peak distribution across genomic loci show close proximity to neighboring genes. The x-axis of the plot represents genes and Y-axis represents the distribution of HDAC6 associated chromatin peaks to map their distribution close to protein coding gene loci. (**C**) The HOMER based motif enrichment analysis identified the binding motif for ZNF264. (**D**) Connectivity of gene ontology (biological process) results based on nearest genes from the region of significant peak change.

ChIP-seq experiments were performed to reveal the genome-wide Hdac6 binding regions in PFC of sham and CORT-treated groups. As shown in Fig. 4A, the ChIP-seq experiment revealed genome-wide binding of Hdac6 largely located in the intergenic region (25/455, 94.5%); rest of them were in the intronic region (25/455, 5.5%). In Fig. 4B, Hdac6 associated peaks and their distribution, proximal to respective genes, have been shown with ridgeline distribution plot. Considering the p-values, the statistically highest known binding motif was common to ZNF264 (Fig. 4C).

### GO analysis based on nearest genes from the region of significant peak change

All 455 genes identified from ChIP-Seq results were subjected to Cytoscape-ClueGO for biological processing (BP) analysis and creating network based on significant BP terms. As shown in Table S4, multiple CNS related BP terms (e.g., synapse organization, dendrite development, morphogenesis, axonogenesis, and neuron projection morphogenesis) were revealed as significant GO terms. These terms had high connectivity among themselves (Fig. 4D).

### Determining the effect of GR in inducing the endogenous expression of miR-218 using in vitro cellular model

The role of GR in transcriptionally activating the expression of miR-218 was tested in a GR overexpressing cell culture system. The in vitro cellular model-based gain of function mutation for NR3C1 gene have supported the induced expression of miR-218. The qPCR results from overexpression experiment demonstrated consistence increase in miR-218 expression (20%) induced by GR as compared to scramble control (Fig. S1).

## Discussion

In this study, we found that CORT enhanced the gene transcription of miR-218-5p, which appeared to be through the biding of GRs on the promoter region of Slit3 gene. Using RNA-IP and input RNA-seq data from miR-218-5p overexpressed SH-SY5Y cells, six genes (APOL4, DTWD1, BNIP1, METLL22, SNAPC1, and HDAC6) were identified as consistent target genes of miR-218-5p. Five genes except APOL4 (not expressed in rats) were downregulated in PFC of CORT-treated rats. Of these, ChIP-seq data in PFC of CORT-treated rats revealed that the nearest genes from Hdac6-associated peaks were associated with several CNS related biological processes terms.

Glucocorticoid receptors are known to activate the transcription of early response genes associated with stress^5,6^. Our aim was to understand the mechanism of upregulated transcriptional activity of miR-218a by CORT. Our results show that mir-218 could possibly be co-activated by CORT, given that miR-218 gene is located in the intron of Slit3 gene. ChIP-seq experiment using GR antibody indicated significant GR binding sites on Slit3 promoter region of CORT-treated rats. We found a trend towards the increased binding of GR on both miR-218a-1 and -2; however, it did not reach statistical significance. This is not surprising given that there are three predicted GR biding sites on Slit3 promoter regions compared to two predicted GR biding sites on other Slit genes. Our in vitro GR overexpression model also supplemented this finding where we found an increased miR-218 expression under exogenous gain of function manipulation of NR3C1 gene following a transfection of cDNA overexpression clone in HEK-293 cell line. It is intriguing that GR mediated gene promoter activity largely depends on interaction with coregulatory molecules. In fact, depending on the nature of the coregulator, GR can either activate or inhibit promoter specific gene transcription. Studies in the past have reported allosteric modulation of GR by transcriptional coregulators, leading to chromatin destabilization and subsequent activation of target gene promoter^29^. Although our ChIP experiment results are not supplemented with coactivator experiments, the present findings related to GR-induced miR-218 expression can be well articulated with the allosteric modulation theory of GR. Despite the mixed findings on stress-induced miR-218 expression changes, our report for the first time elaborated the direct role of GR in transcriptional activation of miR-218 by Slit3 gene promoter. This has also, for the first time, helped in understanding the co-transcriptional activation of an intronic miRNA via a shared promoter usage. Given the role of Slit3 gene in axonal guidance and genetic predisposition to major depressive disorder due its duplicated locus on 5q35.1, it is possible that co-transcriptional activation of Slit3 and miR-218 may be necessary to develop depressive phenotypes under chronic stress^30^.

Since induced transcriptional activity of miRNAs is directly associated with posttranscriptional target gene silencing, we investigated the direct repression of targetome of miR-218-5p using Ago2 mediated RISC-sequencing experiment. There are eight AGO family members, and AGO1-4 are capable of loading miRNA and endonuclease activity. However, RNA interference-dependent gene silencing exclusively belongs to AGO2, which is one of the essential components of RISC. Functionally, mature miRNAs are incorporated into RISC, which helps them regulate the fate of target gene expression by pairing primarily to the 3’-UTR of protein-coding mRNAs. In this context, high throughput RISC profiling is critical to ascertain the role of specific miRNA, in this case miR-218, to determine its regulatory potential on cellular targets critical in stress responsive pathways. To find consistent target genes of miR-218-5p, we conducted RNA-seq with RNA-input samples and found six genes as miR-218-5p consistent target genes. Subsequently, we confirmed the downregulation of five genes (Dtwd1, Bnip1, Mettl22, Snapc1, and Hdac6) by qPCR experiment in PFC of CORT-treated rats. DTWD1 (DTW domain containing 1) is found in STAR*D study as a candidate gene associated with the side effect of antidepressant^31^. Budde et al. reported that rs586758 located in DTWD1 is related to the Global Assessment of Functioning (GAF; DSM-IV Axis V)^32^. Another target gene, BNIP1, is implicated in the functions of the endoplasmic reticulum (ER), which is primarily responsible for cellular stress responses^33^. Under a stressful condition, protein folding is disrupted^34^. ER-mediated unfolded protein response (UPR) is activated to re-establish the protein homeostasis. We have earlier reported the dysregulated expressions of UPR genes in postmortem PFC of MDD subjects^35^ and rat brain which showed depression phenotype^36,37^. The abnormality of UPR genes has also reported in PFC of schizophrenia subjects^38^. METTL22, a member of methyltransferase like protein family, is responsible for the methylation of DNA-RNA-binding protein Kin17 and is implicated in DNA repair and replication and mRNA processing^39^. SNAPC1, one of the subunits of small nuclear RNA (snRNA) activating protein complex, acts as a transcription factor to mediate transcription of snRNAs^40,41^. Interestingly, SNAPC1 acts as a transcriptional coactivator facilitating RNA polymerase II elongating the transcripts^42^.

One of the miR-218-5p target genes is HDAC6, which belongs to epigenetic family of chromatin modifiers. HDAC (histone deacetylase) is a key enzyme that regulates the reversible acetylation of histones^43–45^. Out of four subtypes in HDAC, HDAC6 belongs to Class II. As a direct target of miR-218-5p, Hdac6 can potentially alter genome-wide chromatin conformation of many stress-responsive genes under chronic stress. It would be interesting to know if miR-218 as a posttranscriptional gene regulator can provide an additional layer of epigenetic masking on HDAC6 expression. Our ChIP-seq data obtained from in vitro cell culture model showed that HDAC6 peaks were associated with genes related to key CNS functions including axonal morphogenesis and synaptic functions. Since glucocorticoid signaling system is partly regulated by reversible acetylation-deacetylation level of HSP90 chaperonin molecule^46^, there could be a possibility to see abnormalities in stress responsiveness mediated by altered HDAC6 activity. We strongly anticipate that reduced level of HDAC6 in PFC of rats (as we found in our CORT treated rats) could be part of an epigenetic compensatory mechanism. At the molecular level, this compensation could be attributed to the induced expression of miR-218-5p to directly check the deacetylation status of HSP90 and facilitating other HDAC6 target proteins to achieve a hyperacetylation level under chronic stress^47^. In our ChIP-seq results, we found that the majority of Hdac6 peaks were localized in the intergenic regions (94.5%), as previously reported for other class II HDACs such as HDAC4^48^, HDAC5^49^, HDAC7^50,51^, and HDAC9^48^. The gene ontology results indicated that nearest genes from Hdac6 peak were associated with several CNS related biological processes terms. It has earlier been reported that HDAC6 regulates neural migration and dendrite development^52^, as well as modulates synaptic plasticity and neuronal differentiation in human stem-cell-derived neurons^53^. Especially in PFC of acute stressed rats, HDAC6 regulate the synaptic efficacy^54^. The decreased expression of HDAC6 has also been found in the peripheral blood of bipolar patients^55^. Importantly, HDAC6 inhibitor potentially has antidepressant effect and Hdac6-deficient mice exhibit less anxiety and antidepressant-like behavior^56^.

Altogether, we found that miR-218-5p, upregulated in brain of CORT-treated rats, was GR-mediated. This upregulation, and consequent downregulation of target genes, could potentially be involved in the development of depressive phenotypes in stressed rats. We found 6 consistent target genes of miR-218-5p that were detected by in-vitro experiments in which miR-218-5p was overexpressed followed by RNA-seq. Of them, the nearest genes from HDAC6 peak, identified via ChIP-seq data, were associated with key CNS functions including axonal morphogenesis and synaptic functions. Our ChIP-seq results also identified several HDAC6-associated peaks across the genomic loci, which largely show the involvement of this intragenic miR-218-5p in the overall modification of chromatin sites. Even though the subcellular distribution of HDAC6 is cytoplasmic, it can also shuttle back to the nucleus and may interact with many nuclear proteins^57^. This has been seen as a tight control on chromatin accessibility outlining the importance of reversible chromatin modifications of key stress-associated genes. Together, these findings provide regulatory insights of miR-218-5p, which could possibly be associated with stress-induced depression. It also provides possible feature therapeutic target for the treatment of depression. Further studies in animals overexpressing miR-218, and examining consequent behavioral phenotype, will be key to several of these answers.

## Supplementary Information

Supplementary Figure S1.

Supplementary Table S1.

Supplementary Table S2.

Supplementary Table S3.

Supplementary Table S4.

## Data Availability

The datasets generated during and/or analyzed during the current study are available from the corresponding author on reasonable request.
