# Supplementary figures and images for: Corticosterone-mediated regulation and functions of miR-218-5p in rat brain
Source: Sci Rep. 2022 Jan 7;12:194. doi: 10.1038/s41598-021-03863-y (PMC8742130; doi:10.1038/s41598-021-03863-y)

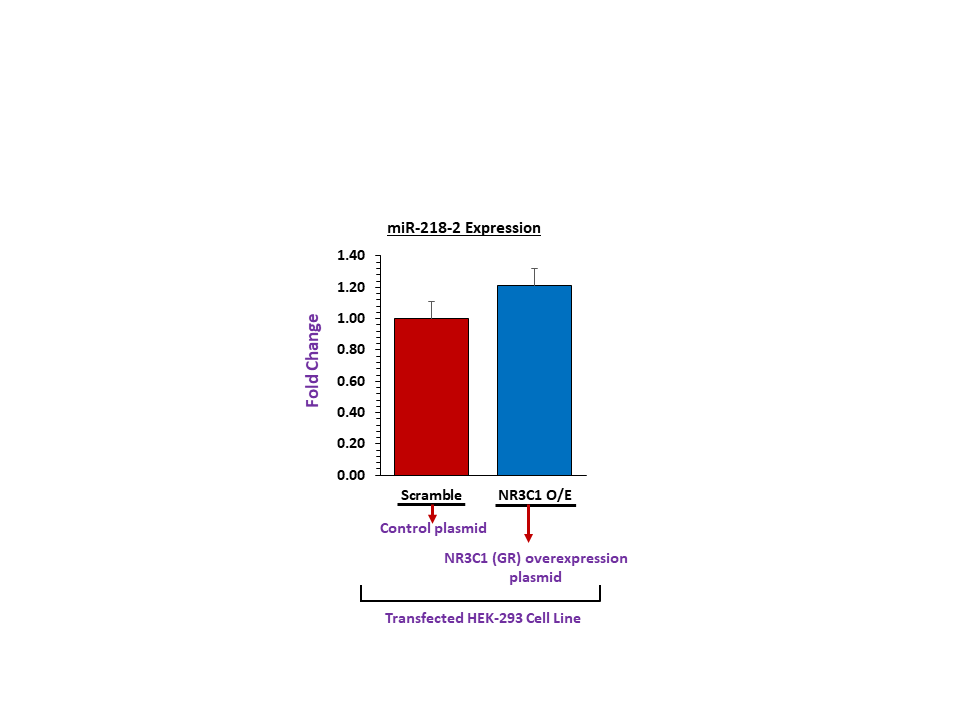

Supplement: Supplementary file 1 — Supplementary Figure S1. [file 41598_2021_3863_MOESM1_ESM.tif]
