# Supplementary material for: Corticosterone-mediated regulation and functions of miR-218-5p in rat brain
Source: Sci Rep. 2022 Jan 7;12:194. doi: 10.1038/s41598-021-03863-y (PMC8742130; doi:10.1038/s41598-021-03863-y)
Supplement: Supplementary file 2 — Supplementary Table S1. [file 41598_2021_3863_MOESM2_ESM.docx]

**Table S1. The prime sequences and thermal cycling parameters for qPCR assays**

| **Primer name** | **Primer Orientation** | **Sequence** | **cDNA dilution** | **Primer concentration**  (µM) | **Thermal parameters** |
| --- | --- | --- | --- | --- | --- |
| **miRNA cDNA synthesis** |  |  |  |  |  |
| Oligo dT Adapter Primer V3 |  | GCGAGCACAGAATTAATACGACTCACTATAGGTTTTTTTTTTTTTTTTTTVN |  |  |  |
|  | | | | | |
| **miRNA qPCR** |  |  |  |  |  |
| hsa_miR-218-5p  rno_miR-218-5p | F | TTGTGCTTGATCTAACCATGTAA | 30x | 0.8 | 95’C for 10 sec, (95’C for 10sec, 55’C for 15 sec, 20 sec for 20 sec) x 40 cycle, 95’C for 1 min, 55’C for 30 sec, 95’C for 30 sec |
| Universal Reverse |  | GCGAGCACAGAATTAATACGAC |  |  |  |
| U6 | F | CTCGCTTCGGCAGCACA | 30x |  |  |
|  | R | AACGCTTCACGAATTTGCGT |  |  |  |
|  | | | | | |
| **mRNA qPCR** |  |  |  |  |  |
| Dtdw1 | F | GTGGACTACCATAGAGCCGTG | 20x | 0.8 | 95’C for 10 sec, (95’C for 10sec, 60’C for 15 sec, 20 sec for 20 sec) x 40 cycle, 95’C for 1 min, 55’C for 30 sec, 95’C for 30 sec |
|  | R | AGAGCGTCTGGCCTCCTTTA |  |  |  |
| Bnip1 | F | GCTGCCACATAGAACACGTC |  |  |  |
|  | R | CCACACCCAGCATTCACCAT |  |  |  |
| Mettl22 | F | GCCATGTGCCAACGAAATGT |  |  |  |
|  | R | TCCTTGACCTTAACCACGCC |  |  |  |
| Snapc1 | F | CTCGGATGGCCTCAGCAAAG |  |  |  |
|  | R | CACCAGTCTCCTGGTTCAGTG |  |  |  |
| Hdac6 | F | CTGCCTGCATGACCGTTCTA |  |  |  |
|  | R | CCACTGCCACTTGTCTCCTT |  |  |  |
| Gapdh | F | CACTGAGCATCTCCCTCACAA |  |  |  |
|  | R | TGGTATTCGAGAGAAGGGAGG |  |  |  |
|  |  |  |  |  |  |
| **ChIP qPCR** |  |  |  |  |  |
| Slit2 promotor | F | TAGAGCGGTCCCCTTTAGGT | 1x | 0.5 | 95’C for 10 sec, (95’C for 10sec, 60’C for 15 sec, 20 sec for 20 sec) x 40 cycle, 95’C for 1 min, 55’C for 30 sec, 95’C for 30 sec |
|  | R | TCCAGTGGAAAAGGAGGAGA |  |  |  |
| Slit3 promotor | F | CATCGGGGTCCAAGTTCTTA |  |  |  |
|  | R | CACAGCAAGGCCAACAATTA |  |  |  |
| miR-218a-1 promotor | F | CCATTATTGGCTTTTCGTTCA |  |  |  |
|  | R | GCAACGGCCTAAAAGATGTG |  |  |  |
| miR-218a-2 promotor | F | TCCTGGTTCAGTATGTAACTGGTG |  |  |  |
|  | R | GGGAGACTCAGCAGGAGAGA |  |  |  |

Primer orientation: Forward primer (F); Reverse primer (R)
