# Supplementary material for: Corticosterone-mediated regulation and functions of miR-218-5p in rat brain
Source: Sci Rep. 2022 Jan 7;12:194. doi: 10.1038/s41598-021-03863-y (PMC8742130; doi:10.1038/s41598-021-03863-y)
Supplement: Supplementary file 3 — Supplementary Table S2. [file 41598_2021_3863_MOESM3_ESM.docx]

**Table S2. All downregulated genes in input RNA-seq result**

| **Gene symbol** | **Ensemble ID** | **Locus** | **Fold change** | **P value** |
| --- | --- | --- | --- | --- |
| **Downregulated genes** | | | | |
| AP000275.2 | ENSG00000265590.9_4 | chr21:33951132-33984591 | 0.180905 | 0.0004807 |
| MPZL1 | ENSG00000197965.11_3 | chr1:167690429-167761156 | 0.1887864 | 0.0008752 |
| ASCL1 | ENSG00000139352.3_2 | chr12:103351464-103354294 | 0.2022087 | 0.023416 |
| CETN2 | ENSG00000147400.8_2 | chrX:151995517-151999321 | 0.2403282 | 0.0028556 |
| GPD1L | ENSG00000152642.10_3 | chr3:32147181-32210205 | 0.2817786 | 0.0027349 |
| SAR1A | ENSG00000079332.14_3 | chr10:71907045-71930279 | 0.2878885 | 8.231E-05 |
| TEAD1 | ENSG00000187079.16_3 | chr11:12696138-12966030 | 0.3091868 | 0.0183628 |
| RBM38 | ENSG00000132819.16_2 | chr20:55966463-55984389 | 0.3173374 | 0.0043662 |
| BST2 | ENSG00000130303.12_3 | chr19:17513748-17516457 | 0.3246793 | 0.0214216 |
| DAND5 | ENSG00000179284.5_3 | chr19:13075973-13085576 | 0.3482238 | 0.0071002 |
| XIAP | ENSG00000101966.12_3 | chrX:122993574-123047829 | 0.3498343 | 0.0002148 |
| NUDT4P1 | ENSG00000177144.7_2 | chr1:145138424-145139613 | 0.3500236 | 0.006471 |
| PAQR4 | ENSG00000162073.13_2 | chr16:3019246-3023490 | 0.3587607 | 0.0099442 |
| NF2 | ENSG00000186575.17_3 | chr22:29999545-30094587 | 0.372742 | 0.0473079 |
| LIMS1 | ENSG00000169756.16_2 | chr2:109150811-109303702 | 0.3804212 | 0.0092564 |
| PPAN-P2RY11 | ENSG00000243207.6_3 | chr19:10216899-10225456 | 0.3811768 | 0.0006183 |
| NEBL | ENSG00000078114.18_3 | chr10:21068902-21463116 | 0.3857501 | 0.0042869 |
| FAM162B | ENSG00000183807.7_2 | chr6:117073360-117086886 | 0.3881591 | 0.019847 |
| SNRPD2 | ENSG00000125743.10_2 | chr19:46190712-46195827 | 0.3918967 | 0.0235815 |
| VOPP1 | ENSG00000154978.12_2 | chr7:55503749-55640681 | 0.3968603 | 0.0035544 |
| RAB26 | ENSG00000167964.12_3 | chr16:2190804-2204166 | 0.4058169 | 0.0143893 |
| ASIC1 | ENSG00000110881.11_2 | chr12:50451331-50477394 | 0.4111589 | 0.0026826 |
| GADD45G | ENSG00000130222.10_2 | chr9:92219928-92221470 | 0.4112047 | 0.0191962 |
| MSANTD3-TMEFF1 | ENSG00000251349.3_3 | chr9:103204553-103339918 | 0.4139773 | 0.0409161 |
| BCAT1 | ENSG00000060982.14_2 | chr12:24962956-25102393 | 0.4183157 | 0.0049242 |
| PPP1CB | ENSG00000213639.9_3 | chr2:28974506-29025806 | 0.4216363 | 0.0068819 |
| TMEM254 | ENSG00000133678.13_3 | chr10:81838402-81852313 | 0.4225949 | 0.0297372 |
| VIPR2 | ENSG00000106018.13_2 | chr7:158820866-158937649 | 0.4283776 | 0.0035003 |
| C5orf24 | ENSG00000181904.8_3 | chr5:134181370-134195427 | 0.4288526 | 0.043961 |
| SSPN | ENSG00000123096.11_3 | chr12:26274924-26452223 | 0.4321631 | 0.0158153 |
| VAMP7 | ENSG00000124333.15_2 | chrX:155110956-155173433 | 0.4328983 | 0.0262221 |
| MRPL19 | ENSG00000115364.13_3 | chr2:75873909-75917977 | 0.4501348 | 0.0072044 |
| VBP1 | ENSG00000155959.10_2 | chrX:154425284-154468098 | 0.4501619 | 0.0157633 |
| C11orf24 | ENSG00000171067.10_3 | chr11:68028803-68039469 | 0.4531027 | 0.0030246 |
| ARFIP1 | ENSG00000164144.15_3 | chr4:153701089-153839615 | 0.4541119 | 0.0384391 |
| FGFBP2 | ENSG00000137441.7_3 | chr4:15961866-15970932 | 0.4553727 | 0.0012055 |
| GNAI2 | ENSG00000114353.16_3 | chr3:50263724-50296787 | 0.4626822 | 0.0081994 |
| GJA1 | ENSG00000152661.7_2 | chr6:121756838-121770873 | 0.4649476 | 0.0225201 |
| CCDC6 | ENSG00000108091.10_2 | chr10:61548521-61666414 | 0.4652203 | 0.0137334 |
| IL4R | ENSG00000077238.13_3 | chr16:27324989-27376099 | 0.4653786 | 0.0326701 |
| COX4I2 | ENSG00000131055.4_2 | chr20:30225691-30232809 | 0.4663231 | 0.0251944 |
| RPP25 | ENSG00000178718.6_2 | chr15:75246757-75249805 | 0.4729804 | 0.0081071 |
| PTP4A3 | ENSG00000184489.11_4 | chr8:142402093-142442554 | 0.4733279 | 0.0419823 |
| ZNF385A | ENSG00000161642.17_2 | chr12:54762917-54785082 | 0.4750638 | 0.0087328 |
| CHGA | ENSG00000100604.12_4 | chr14:93389425-93401638 | 0.4826881 | 0.0104454 |
| TMX1 | ENSG00000139921.12_2 | chr14:51706880-51724264 | 0.4875876 | 0.043649 |
| SSTR2 | ENSG00000180616.8_3 | chr17:71161151-71172772 | 0.4899205 | 0.0323106 |
| LYRM7 | ENSG00000186687.15_2 | chr5:130506503-130541119 | 0.49913 | 0.0107897 |
| SV2A | ENSG00000159164.9_2 | chr1:149874870-149889434 | 0.4997231 | 0.0025381 |
| CBS | ENSG00000160200.17_2 | chr21:44473301-44497053 | 0.5023665 | 0.0013395 |
| THAP5 | ENSG00000177683.13_3 | chr7:108194987-108210194 | 0.504403 | 0.0130431 |
| RIMS3 | ENSG00000117016.9_2 | chr1:41086351-41131329 | 0.5085239 | 0.0246419 |
| CCNI | ENSG00000118816.9_3 | chr4:77968311-77997158 | 0.5124011 | 0.000891 |
| GMNN | ENSG00000112312.9_3 | chr6:24775159-24786327 | 0.5151641 | 0.0255806 |
| TPRG1L | ENSG00000158109.14_2 | chr1:3541566-3546691 | 0.5200782 | 0.0077036 |
| PPP1CC | ENSG00000186298.11_2 | chr12:111157485-111180744 | 0.5227236 | 0.0201723 |
| E2F2 | ENSG00000007968.6_2 | chr1:23832922-23857712 | 0.5262375 | 0.0360094 |
| ANKRD40 | ENSG00000154945.6_2 | chr17:48770551-48785285 | 0.5283676 | 0.0124219 |
| PSMB8 | ENSG00000204264.8_2 | chr6:32808494-32812480 | 0.530056 | 0.0001688 |
| PAIP2 | ENSG00000120727.12_3 | chr5:138677276-138705409 | 0.5304938 | 0.0050067 |
| GLTP | ENSG00000139433.9_3 | chr12:110288748-110318293 | 0.5354408 | 0.0035472 |
| SLC44A3 | ENSG00000143036.16_2 | chr1:95285898-95360802 | 0.5355304 | 0.0213842 |
| TNIP2 | ENSG00000168884.14_3 | chr4:2743375-2758103 | 0.5360018 | 0.0387046 |
| SAMHD1 | ENSG00000101347.8_2 | chr20:35518632-35580246 | 0.539487 | 0.0345441 |
| FTH1 | ENSG00000167996.15_3 | chr11:61727190-61735132 | 0.5405658 | 0.0074584 |
| SLC29A3 | ENSG00000198246.7_2 | chr10:73079015-73123142 | 0.5412389 | 0.000617 |
| CNTNAP2 | ENSG00000174469.19_3 | chr7:145813094-148118090 | 0.5419532 | 0.0131425 |
| CDCA7L | ENSG00000164649.19_3 | chr7:21940518-21985702 | 0.5424992 | 0.0469496 |
| NPPA | ENSG00000175206.10_2 | chr1:11905766-11908402 | 0.5446883 | 0.0011187 |
| SERPINF1 | ENSG00000132386.10_2 | chr17:1665253-1680868 | 0.5454086 | 0.020596 |
| PUM2 | ENSG00000055917.15_2 | chr2:20448452-20551995 | 0.5457828 | 0.0201167 |
| ANKRD1 | ENSG00000148677.6_2 | chr10:92671853-92681033 | 0.5459934 | 0.0303593 |
| FOLR1 | ENSG00000110195.12_2 | chr11:71900602-71907367 | 0.5472608 | 0.0224136 |
| FBP1 | ENSG00000165140.9_2 | chr9:97365415-97402531 | 0.5524894 | 0.0330408 |
| UBE2G2 | ENSG00000184787.18_2 | chr21:46188495-46221934 | 0.5567653 | 0.0263405 |
| NDUFA6 | ENSG00000184983.9_3 | chr22:42481529-42486959 | 0.5586537 | 0.0125828 |
| SSR3 | ENSG00000114850.6_2 | chr3:156257929-156272973 | 0.558929 | 0.0079325 |
| RRP1B | ENSG00000160208.12_2 | chr21:45079429-45115960 | 0.5608973 | 0.0007939 |
| SH3GL1 | ENSG00000141985.9_3 | chr19:4360367-4400544 | 0.5634921 | 0.0456188 |
| PLGRKT | ENSG00000107020.9_3 | chr9:5357973-5437878 | 0.5639747 | 0.0432228 |
| AL136295.5 | ENSG00000259529.1_4 | chr14:24620427-24636611 | 0.5647468 | 0.0076918 |
| MARCKS | ENSG00000277443.2_3 | chr6:114178541-114184648 | 0.566794 | 0.0034756 |
| HRK | ENSG00000135116.9_3 | chr12:117293949-117319246 | 0.5684322 | 0.0243191 |
| MXI1 | ENSG00000119950.20_4 | chr10:111967363-112047123 | 0.5719332 | 0.0337033 |
| TRAK2 | ENSG00000115993.12_3 | chr2:202241930-202316302 | 0.5731084 | 0.0066372 |
| AMMECR1 | ENSG00000101935.9_3 | chrX:109437414-109683461 | 0.57532 | 0.0422492 |
| UBE2D4 | ENSG00000078967.12_3 | chr7:43966037-43995735 | 0.5791508 | 0.0008656 |
| TMEM53 | ENSG00000126106.13_2 | chr1:45100910-45140227 | 0.5799841 | 0.0300517 |
| DCBLD2 | ENSG00000057019.15_3 | chr3:98514785-98620533 | 0.5805079 | 0.0028628 |
| DLL3 | ENSG00000090932.10_2 | chr19:39989535-39999121 | 0.5821268 | 0.0005202 |
| MGEA5 | ENSG00000198408.13_2 | chr10:103544200-103578222 | 0.5836239 | 0.0057907 |
| FAM172A | ENSG00000113391.18_3 | chr5:92953775-93447404 | 0.5843074 | 0.0150966 |
| NACA | ENSG00000196531.10_3 | chr12:57106212-57125412 | 0.586717 | 0.0365699 |
| PNPLA2 | ENSG00000177666.16_3 | chr11:818902-825573 | 0.5867889 | 0.0446926 |
| FAM20B | ENSG00000116199.11_2 | chr1:178994939-179045697 | 0.592527 | 0.0365565 |
| RPS6KA6 | ENSG00000072133.10_2 | chrX:83313354-83442915 | 0.5937778 | 0.0085835 |
| FOXN3 | ENSG00000053254.15_3 | chr14:89591215-90085493 | 0.5952694 | 0.0150586 |
| ID1 | ENSG00000125968.8_2 | chr20:30193086-30194318 | 0.5998687 | 0.0348611 |
| TLE6 | ENSG00000104953.19_3 | chr19:2977444-2995182 | 0.6005 | 0.0101107 |
| HIST1H4J | ENSG00000197238.4_2 | chr6:27791886-27792258 | 0.6012629 | 0.02786 |
| ABHD14A | ENSG00000248487.8_3 | chr3:52005442-52015215 | 0.6050124 | 0.0251241 |
| CYGB | ENSG00000161544.9_3 | chr17:74523438-74547257 | 0.605323 | 0.0285486 |
| HIST2H2AA4 | ENSG00000203812.2 | chr1:149822643-149823191 | 0.6059559 | 0.0076381 |
| TMEM119 | ENSG00000183160.8_4 | chr12:108983622-108992096 | 0.6060002 | 0.0138633 |
| OBP2A | ENSG00000122136.13_2 | chr9:138437985-138441815 | 0.609037 | 0.0094865 |
| DDX56 | ENSG00000136271.10_3 | chr7:44605016-44614650 | 0.6098479 | 0.0405555 |
| HIST1H4K | ENSG00000273542.1_2 | chr6:27798994-27799305 | 0.6129612 | 0.0460201 |
| CACUL1 | ENSG00000151893.14_3 | chr10:120433679-120514761 | 0.6138731 | 0.008386 |
| BEX1 | ENSG00000133169.5_2 | chrX:102317579-102319168 | 0.6147046 | 0.0478195 |
| GDE1 | ENSG00000006007.11_2 | chr16:19513011-19533467 | 0.6149608 | 0.0199653 |
| SYCE3 | ENSG00000217442.3_2 | chr22:50989541-51001334 | 0.6170111 | 0.0416849 |
| YEATS4 | ENSG00000127337.6_2 | chr12:69753483-69784576 | 0.6185343 | 0.0055062 |
| GPR161 | ENSG00000143147.14_2 | chr1:168048781-168106905 | 0.6190164 | 0.0250432 |
| EZR | ENSG00000092820.17_2 | chr6:159186773-159240444 | 0.6216591 | 0.0388033 |
| SMIM18 | ENSG00000253457.2_2 | chr8:30496117-30503581 | 0.6227888 | 0.0130925 |
| FLRT1 | ENSG00000126500.3_3 | chr11:63870660-63886645 | 0.6233332 | 0.0187525 |
| RIN1 | ENSG00000174791.10_2 | chr11:66097713-66104311 | 0.6252449 | 0.0239632 |
| HIST2H2AA3 | ENSG00000183558.5 | chr1:149813505-149814478 | 0.6276789 | 0.0062325 |
| IMPAD1 | ENSG00000104331.8_4 | chr8:57870492-57906403 | 0.6277244 | 0.0248417 |
| DDX21 | ENSG00000165732.12_2 | chr10:70715884-70744829 | 0.6282422 | 0.0460025 |
| FGL1 | ENSG00000104760.16_2 | chr8:17721889-17767874 | 0.6287989 | 0.0116722 |
| EMID1 | ENSG00000186998.15_2 | chr22:29601840-29655586 | 0.6304217 | 0.0386212 |
| BIRC5 | ENSG00000089685.14_3 | chr17:76210267-76221717 | 0.6326102 | 0.0176822 |
| ADIPOR2 | ENSG00000006831.9_2 | chr12:1797740-1897844 | 0.6352321 | 0.029305 |
| PRKX | ENSG00000183943.5_2 | chrX:3522411-3631649 | 0.6355843 | 0.0004685 |
| HPCAL4 | ENSG00000116983.12_2 | chr1:40144320-40157361 | 0.6360048 | 0.0450234 |
| KCTD16 | ENSG00000183775.10_3 | chr5:143550396-143865249 | 0.6363869 | 0.003341 |
| NR1D2 | ENSG00000174738.12_2 | chr3:23986751-24022109 | 0.6372336 | 0.0273004 |
| C20orf27 | ENSG00000101220.17_2 | chr20:3734155-3749034 | 0.6374175 | 0.0457914 |
| MEPCE | ENSG00000146834.13_2 | chr7:100026413-100031749 | 0.6379201 | 0.0149702 |
| PARP12 | ENSG00000059378.12_2 | chr7:139723544-139763521 | 0.6383449 | 0.0010512 |
| AKR1A1 | ENSG00000117448.13_3 | chr1:46016215-46035721 | 0.6393635 | 0.0339059 |
| RNF149 | ENSG00000163162.8_2 | chr2:101887681-101925163 | 0.640129 | 0.0045854 |
| ZDHHC9 | ENSG00000188706.12_2 | chrX:128937264-128977885 | 0.6420979 | 0.0072646 |
| NRP2 | ENSG00000118257.16_3 | chr2:206546714-206662857 | 0.6425582 | 0.0162046 |
| PEX11G | ENSG00000104883.7_3 | chr19:7541761-7562335 | 0.6444377 | 0.0370133 |
| EIF2S3 | ENSG00000130741.10_2 | chrX:24072833-24096088 | 0.6461336 | 0.0003728 |
| GSKIP | ENSG00000100744.14_2 | chr14:96829789-96853625 | 0.6473385 | 0.032026 |
| WFS1 | ENSG00000109501.13_3 | chr4:6271576-6304992 | 0.6503047 | 0.0232083 |
| F12 | ENSG00000131187.9_2 | chr5:176829141-176836577 | 0.651407 | 0.02456 |
| NLE1 | ENSG00000073536.17_2 | chr17:33455772-33469334 | 0.6535545 | 0.0476169 |
| CTDNEP1 | ENSG00000175826.11_3 | chr17:7146910-7155810 | 0.653607 | 0.0097865 |
| POLR2C | ENSG00000102978.12_3 | chr16:57496299-57505922 | 0.6547756 | 0.0398617 |
| MRPL11 | ENSG00000174547.13_4 | chr11:66202546-66234209 | 0.6555947 | 0.0242304 |
| RANBP3 | ENSG00000031823.14_3 | chr19:5916150-5978153 | 0.6566381 | 0.0061123 |
| LFNG | ENSG00000106003.12_2 | chr7:2552163-2568811 | 0.6575834 | 0.0162623 |
| RPL22L1 | ENSG00000163584.17_2 | chr3:170582664-170588272 | 0.6578675 | 0.0043175 |
| TAOK3 | ENSG00000135090.13_3 | chr12:118587606-118810750 | 0.6582118 | 0.021339 |
| GLCE | ENSG00000138604.9_3 | chr15:69452923-69564556 | 0.6594151 | 0.0098339 |
| NGDN | ENSG00000129460.15_4 | chr14:23938897-23979071 | 0.6597353 | 0.0101161 |
| THSD7A | ENSG00000005108.15_3 | chr7:11409984-11871824 | 0.6601822 | 0.0177923 |
| BUB3 | ENSG00000154473.17_2 | chr10:124913793-124929983 | 0.6604391 | 0.0444269 |
| THBS3 | ENSG00000169231.13_2 | chr1:155165379-155178842 | 0.6605241 | 0.0321205 |
| DHDH | ENSG00000104808.7_2 | chr19:49436939-49448226 | 0.6615207 | 0.0169186 |
| SNUPN | ENSG00000169371.13_3 | chr15:75890424-75918810 | 0.6615268 | 0.0424014 |
| CELF1 | ENSG00000149187.18_4 | chr11:47487485-47587121 | 0.6615272 | 0.0432528 |
| SLC39A3 | ENSG00000141873.10_3 | chr19:2732202-2740150 | 0.6622962 | 0.0238401 |
| METTL16 | ENSG00000127804.12_3 | chr17:2308856-2415185 | 0.6634376 | 0.0014502 |
| FAM98A | ENSG00000119812.18_3 | chr2:33808725-33824449 | 0.6652136 | 0.0128103 |
| MAGT1 | ENSG00000102158.19_2 | chrX:77081861-77151090 | 0.6652571 | 0.0093406 |
| HERC6 | ENSG00000138642.14_3 | chr4:89299891-89364263 | 0.6652609 | 0.0038379 |
| KIAA1161 | ENSG00000164976.8_2 | chr9:34366668-34376851 | 0.665625 | 0.0287266 |
| AGMAT | ENSG00000116771.5_2 | chr1:15898848-15911605 | 0.6680057 | 0.0250153 |
| TIMP3 | ENSG00000100234.11_2 | chr22:33197687-33259030 | 0.6682866 | 0.0402385 |
| ARL14EP | ENSG00000152219.4_2 | chr11:30344598-30359774 | 0.6687076 | 0.00018 |
| IL32 | ENSG00000008517.16_4 | chr16:3115298-3132193 | 0.6706384 | 0.0067594 |
| FABP5 | ENSG00000164687.10_2 | chr8:82192598-82197012 | 0.6708791 | 0.0258594 |
| HIST1H2AE | ENSG00000277075.2_2 | chr6:26217203-26217711 | 0.6720311 | 0.0158069 |
| CNRIP1 | ENSG00000119865.8_2 | chr2:68511303-68547183 | 0.6720409 | 0.0370646 |
| SIL1 | ENSG00000120725.12_3 | chr5:138282409-138629246 | 0.6732885 | 0.0384477 |
| BNC2 | ENSG00000173068.17_3 | chr9:16409501-16870841 | 0.6733762 | 0.0007402 |
| APOL4 | ENSG00000100336.17_3 | chr22:36585172-36600886 | 0.6750032 | 0.0292103 |
| CCDC110 | ENSG00000168491.9_3 | chr4:186366336-186392913 | 0.6750131 | 0.043003 |
| SSR1 | ENSG00000124783.12_3 | chr6:7268539-7347679 | 0.6760898 | 0.0368776 |
| BTBD2 | ENSG00000133243.8_3 | chr19:1985437-2034880 | 0.6770427 | 0.0172141 |
| FUNDC2 | ENSG00000165775.17_2 | chrX:154254255-154288578 | 0.6777337 | 0.0026844 |
| ARAF | ENSG00000078061.12_2 | chrX:47420499-47431319 | 0.6793089 | 0.0482225 |
| WARS2 | ENSG00000116874.11_3 | chr1:119573839-119683294 | 0.6802892 | 0.0033707 |
| ILVBL | ENSG00000105135.15_2 | chr19:15225795-15236596 | 0.6805193 | 0.0321245 |
| GGT2 | ENSG00000133475.17_4 | chr22:21562262-21581926 | 0.6809856 | 0.0160437 |
| ZNF556 | ENSG00000172000.7_2 | chr19:2867333-2883443 | 0.6812091 | 0.0160242 |
| COTL1 | ENSG00000103187.7_2 | chr16:84599200-84651683 | 0.6839259 | 0.040691 |
| TSR1 | ENSG00000167721.10_3 | chr17:2225797-2240801 | 0.6852454 | 0.0036024 |
| HIST3H2A | ENSG00000181218.5_2 | chr1:228644680-228645574 | 0.6854401 | 0.0346966 |
| ATIC | ENSG00000138363.14_3 | chr2:216176540-216214496 | 0.6855215 | 0.0373234 |
| SHMT1 | ENSG00000176974.19_3 | chr17:18231174-18266877 | 0.6857134 | 0.0233031 |
| TBC1D24 | ENSG00000162065.12_3 | chr16:2525052-2559561 | 0.68694 | 0.011327 |
| FRMD3 | ENSG00000172159.15_2 | chr9:85857905-86153461 | 0.6875824 | 0.0011382 |
| RBCK1 | ENSG00000125826.20_3 | chr20:388142-411610 | 0.6880813 | 0.0235526 |
| ZNF704 | ENSG00000164684.13_3 | chr8:81540686-81787016 | 0.6889073 | 0.0042962 |
| TM4SF4 | ENSG00000169903.6_3 | chr3:149191761-149221068 | 0.689889 | 0.0090053 |
| SERPINI1 | ENSG00000163536.12_2 | chr3:167453031-167543356 | 0.6904628 | 0.0329487 |
| CYP51A1 | ENSG00000001630.15_3 | chr7:91741465-91772266 | 0.6910578 | 0.0443503 |
| ARRB2 | ENSG00000141480.17_2 | chr17:4613784-4624794 | 0.6912741 | 0.0141088 |
| MRM1 | ENSG00000278619.4_2 | chr17:34958001-34965407 | 0.691276 | 0.033481 |
| JMY | ENSG00000152409.8_2 | chr5:78532012-78623038 | 0.6914695 | 0.0436536 |
| DPAGT1 | ENSG00000172269.18_3 | chr11:118967213-118979041 | 0.6941191 | 0.0202077 |
| SLC39A4 | ENSG00000147804.9_3 | chr8:145635126-145642279 | 0.6967241 | 0.0066411 |
| CCNY | ENSG00000108100.17_2 | chr10:35535953-35861597 | 0.6986836 | 0.0008653 |
| WDFY1 | ENSG00000085449.14_3 | chr2:224720433-224810104 | 0.6993681 | 0.0191374 |
| AKR1B1 | ENSG00000085662.13_2 | chr7:134127102-134144036 | 0.7022938 | 0.0089535 |
| SLC27A5 | ENSG00000083807.9_4 | chr19:58990879-59023780 | 0.7023502 | 0.0481141 |
| DTWD1 | ENSG00000104047.14_2 | chr15:49913177-49948429 | 0.7038871 | 0.0100971 |
| TXNL4A | ENSG00000141759.14_2 | chr18:77730811-77793949 | 0.7048269 | 0.0018518 |
| LMF2 | ENSG00000100258.17_3 | chr22:50941378-50946120 | 0.7064639 | 0.0196549 |
| ALG1L | ENSG00000189366.9_2 | chr3:125648118-125655882 | 0.7068887 | 0.0272072 |
| ADGRL1 | ENSG00000072071.16_2 | chr19:14258555-14316999 | 0.708029 | 0.0342201 |
| BEND3 | ENSG00000178409.13_2 | chr6:107386386-107436473 | 0.7082166 | 0.0461435 |
| ELOVL2 | ENSG00000197977.3_2 | chr6:10980992-11044547 | 0.7087281 | 0.0404702 |
| CD248 | ENSG00000174807.3_2 | chr11:66081958-66084515 | 0.7089409 | 0.0009237 |
| TPRN | ENSG00000176058.11_3 | chr9:140086069-140098645 | 0.7089648 | 0.0299416 |
| DTX3L | ENSG00000163840.9_2 | chr3:122283085-122294050 | 0.7093263 | 0.0081891 |
| GFRA3 | ENSG00000146013.10_3 | chr5:137588068-137610360 | 0.7111685 | 0.0431218 |
| DNPH1 | ENSG00000112667.12_3 | chr6:43193367-43197222 | 0.7112192 | 0.0142668 |
| LGALSL | ENSG00000119862.12_3 | chr2:64681103-64688515 | 0.712455 | 0.0311742 |
| RUNDC3A | ENSG00000108309.13_2 | chr17:42385781-42396039 | 0.7130642 | 0.0213737 |
| HIST1H2AC | ENSG00000180573.9_2 | chr6:26124373-26139344 | 0.7144721 | 0.036698 |
| CENPB | ENSG00000125817.7_2 | chr20:3764498-3767337 | 0.7147554 | 0.0070418 |
| LETM1 | ENSG00000168924.14_2 | chr4:1813206-1857974 | 0.715935 | 0.0178466 |
| ZNF420 | ENSG00000197050.10_3 | chr19:37498759-37621216 | 0.7178388 | 0.000104 |
| LUZP2 | ENSG00000187398.11_4 | chr11:24518516-25104177 | 0.7179877 | 0.004403 |
| EXOSC3 | ENSG00000107371.12_3 | chr9:37766975-37801434 | 0.7192416 | 0.0140668 |
| LPIN1 | ENSG00000134324.11_3 | chr2:11817721-11967535 | 0.7217224 | 0.0425994 |
| SPCS1 | ENSG00000114902.13_2 | chr3:52738971-52745162 | 0.7219779 | 0.0266239 |
| ZNF592 | ENSG00000166716.9_3 | chr15:85291866-85349663 | 0.7233539 | 0.0019681 |
| TCHP | ENSG00000139437.17_3 | chr12:110338069-110421646 | 0.7238104 | 0.0164046 |
| SLC27A1 | ENSG00000130304.16_3 | chr19:17579578-17616977 | 0.7244909 | 0.0179292 |
| FAM120C | ENSG00000184083.11_3 | chrX:54094757-54209714 | 0.7249998 | 0.0019103 |
| ZNF426 | ENSG00000130818.11_3 | chr19:9633900-9649321 | 0.7253103 | 0.0442171 |
| TMEM39B | ENSG00000121775.17_2 | chr1:32537632-32568467 | 0.7256683 | 0.03603 |
| ZBTB7C | ENSG00000184828.9_3 | chr18:45553044-45937123 | 0.7270464 | 0.0235924 |
| LRBA | ENSG00000198589.10_3 | chr4:151185683-151936879 | 0.7286265 | 0.0137409 |
| CNNM1 | ENSG00000119946.10_2 | chr10:101088856-101154087 | 0.7287669 | 0.048387 |
| LAMP5 | ENSG00000125869.9_2 | chr20:9495005-9511171 | 0.7291262 | 0.0128383 |
| MRPS10 | ENSG00000048544.5_2 | chr6:42174539-42185603 | 0.7296106 | 0.0075397 |
| THEM4 | ENSG00000159445.12_2 | chr1:151846060-151882284 | 0.7299252 | 0.0383912 |
| GPX7 | ENSG00000116157.5_2 | chr1:53068044-53074723 | 0.7305593 | 0.0142827 |
| DNMT3A | ENSG00000119772.16_2 | chr2:25450724-25565459 | 0.730946 | 0.0109616 |
| DMWD | ENSG00000185800.11_3 | chr19:46286205-46296060 | 0.7335196 | 0.0259538 |
| GLA | ENSG00000102393.9_2 | chrX:100652791-100662913 | 0.7339129 | 0.0418622 |
| FEM1A | ENSG00000141965.4_2 | chr19:4791693-4801285 | 0.7342971 | 0.017729 |
| MEA1 | ENSG00000124733.3_2 | chr6:42979832-42981706 | 0.7351918 | 0.0140444 |
| ATP6V1E2 | ENSG00000250565.6_3 | chr2:46717889-46769696 | 0.735229 | 0.0169687 |
| TNNT2 | ENSG00000118194.18_3 | chr1:201328136-201346890 | 0.7362915 | 0.0303412 |
| POGZ | ENSG00000143442.21_3 | chr1:151375200-151431941 | 0.7376831 | 0.0247596 |
| SMIM19 | ENSG00000176209.11_2 | chr8:42396298-42410336 | 0.7381754 | 0.0072133 |
| RABEP2 | ENSG00000177548.12_3 | chr16:28915742-28947847 | 0.7386204 | 0.0342326 |
| DHRS13 | ENSG00000167536.13_2 | chr17:27224799-27230089 | 0.7386508 | 0.0284886 |
| NFE2L2 | ENSG00000116044.15_3 | chr2:178092323-178257425 | 0.7408539 | 0.0246259 |
| KIAA1614 | ENSG00000135835.11_2 | chr1:180882290-180920750 | 0.7418051 | 0.0316021 |
| TMPO | ENSG00000120802.13_3 | chr12:98909290-98944157 | 0.7441681 | 0.0305795 |
| NEUROD1 | ENSG00000162992.3_3 | chr2:182537815-182545603 | 0.7446328 | 0.0374929 |
| SYS1 | ENSG00000204070.9_3 | chr20:43990577-44005438 | 0.7446756 | 0.0143042 |
| ATOH8 | ENSG00000168874.12_2 | chr2:85978467-86015189 | 0.7447817 | 0.0069319 |
| HGH1 | ENSG00000235173.6_2 | chr8:145192672-145195746 | 0.7465082 | 0.0493672 |
| MRPS22 | ENSG00000175110.11_3 | chr3:138724648-139076065 | 0.7474364 | 0.0439497 |
| RPS7 | ENSG00000171863.12_2 | chr2:3622795-3628509 | 0.7486316 | 0.0226187 |
| CLOCK | ENSG00000134852.14_3 | chr4:56294070-56413305 | 0.7497818 | 0.0169778 |
| KEAP1 | ENSG00000079999.13_3 | chr19:10596796-10614417 | 0.7500502 | 0.0499497 |
| IBA57 | ENSG00000181873.12_2 | chr1:228353516-228369958 | 0.750879 | 0.0279234 |
| ARHGAP4 | ENSG00000089820.15_3 | chrX:153172821-153200452 | 0.7518086 | 0.0148709 |
| PODXL2 | ENSG00000114631.10_3 | chr3:127348024-127391652 | 0.7521399 | 0.0476933 |
| ARSJ | ENSG00000180801.13_3 | chr4:114821440-114900883 | 0.7523752 | 0.0324669 |
| RTL5 | ENSG00000242732.4_3 | chrX:71346960-71351751 | 0.7529755 | 0.0025733 |
| ASNA1 | ENSG00000198356.11_2 | chr19:12847953-12859137 | 0.7540725 | 0.0031837 |
| NIFK | ENSG00000155438.11_3 | chr2:122484521-122494499 | 0.7543088 | 0.0439931 |
| DRG2 | ENSG00000108591.9_3 | chr17:17991200-18011285 | 0.7543951 | 0.0291997 |
| ADM | ENSG00000148926.9_3 | chr11:10326227-10328944 | 0.7545141 | 0.0481562 |
| NACC1 | ENSG00000160877.5_3 | chr19:13228917-13251955 | 0.7547451 | 0.0330826 |
| MDP1 | ENSG00000213920.8_3 | chr14:24683143-24685276 | 0.7587552 | 0.032079 |
| PPM1F | ENSG00000100034.13_3 | chr22:22273793-22307209 | 0.759378 | 0.0357308 |
| TSPAN6 | ENSG00000000003.14_2 | chrX:99882106-99894988 | 0.7599079 | 0.0039715 |
| PPP2R5A | ENSG00000066027.11_3 | chr1:212458879-212535205 | 0.7613085 | 0.0297539 |
| CHD6 | ENSG00000124177.14_3 | chr20:40030741-40247133 | 0.7616605 | 0.0032961 |
| TBL2 | ENSG00000106638.15_2 | chr7:72983262-72993121 | 0.7631796 | 0.0378965 |
| PCDH17 | ENSG00000118946.11_2 | chr13:58205944-58303445 | 0.7634424 | 0.0441998 |
| NAPEPLD | ENSG00000161048.11_3 | chr7:102740223-102790007 | 0.763584 | 0.008158 |
| RAD1 | ENSG00000113456.18_2 | chr5:34905369-34919094 | 0.7636138 | 0.012033 |
| PARP14 | ENSG00000173193.13_3 | chr3:122399465-122449687 | 0.7673623 | 0.0036221 |
| ZFAND1 | ENSG00000104231.10_3 | chr8:82613569-82645138 | 0.7675578 | 0.0319661 |
| TXLNA | ENSG00000084652.15_2 | chr1:32645287-32663886 | 0.7692883 | 0.0477871 |
| GTPBP4 | ENSG00000107937.18_2 | chr10:1033959-1065876 | 0.7701063 | 0.0475203 |
| BNIP1 | ENSG00000113734.17_3 | chr5:172571445-172591390 | 0.7708964 | 0.0319268 |
| BAIAP2 | ENSG00000175866.15_3 | chr17:79008922-79091232 | 0.7715444 | 0.009662 |
| CARMIL1 | ENSG00000079691.17_3 | chr6:25279306-25620758 | 0.7730911 | 0.0479823 |
| RAB31 | ENSG00000168461.12_3 | chr18:9708162-9862548 | 0.7750302 | 0.0079823 |
| FBRSL1 | ENSG00000112787.12_3 | chr12:133066137-133161774 | 0.7759615 | 0.0451779 |
| POPDC2 | ENSG00000121577.13_3 | chr3:119355304-119384171 | 0.7779009 | 0.0128187 |
| SECTM1 | ENSG00000141574.7_4 | chr17:80278900-80291950 | 0.7789091 | 0.0481482 |
| BTBD11 | ENSG00000151136.14_3 | chr12:107712190-108053419 | 0.7823737 | 0.0303426 |
| ZNF613 | ENSG00000176024.17_3 | chr19:52430400-52452012 | 0.7824093 | 0.0249224 |
| MON1B | ENSG00000103111.14_3 | chr16:77224732-77236302 | 0.7829688 | 0.0055609 |
| DEPTOR | ENSG00000155792.9_2 | chr8:120885957-121063152 | 0.7833474 | 0.0428688 |
| AP3B2 | ENSG00000103723.12_3 | chr15:83328033-83378666 | 0.7848637 | 0.0335367 |
| PHF21B | ENSG00000056487.15_3 | chr22:45277042-45405880 | 0.7855487 | 0.0436967 |
| POU6F1 | ENSG00000184271.16_3 | chr12:51580719-51611492 | 0.7856601 | 0.0318556 |
| SF1 | ENSG00000168066.20_3 | chr11:64532078-64546258 | 0.7859193 | 0.0310563 |
| PGAP3 | ENSG00000161395.13_3 | chr17:37827375-37853050 | 0.7869277 | 0.0375739 |
| ETV4 | ENSG00000175832.12_3 | chr17:41605212-41656988 | 0.788658 | 0.032104 |
| SNAP29 | ENSG00000099940.11_3 | chr22:21213271-21245506 | 0.7903001 | 0.0195069 |
| CCDC74B | ENSG00000152076.18_2 | chr2:130896860-130902707 | 0.7914557 | 0.0269022 |
| SEMA5A | ENSG00000112902.11_3 | chr5:9035138-9546187 | 0.7919325 | 0.0436697 |
| FZD2 | ENSG00000180340.6_2 | chr17:42634827-42636938 | 0.7920384 | 0.0162918 |
| LSP1 | ENSG00000130592.15_1 | chr11:1874200-1913497 | 0.7998622 | 0.0431689 |
| CCT8 | ENSG00000156261.12_3 | chr21:30428126-30446118 | 0.8008784 | 0.0388206 |
| ACSF3 | ENSG00000176715.15_3 | chr16:89154783-89222254 | 0.801458 | 0.028482 |
| SSTR3 | ENSG00000278195.1_3 | chr22:37600278-37608362 | 0.8018006 | 0.0357601 |
| LARP4B | ENSG00000107929.14_2 | chr10:852854-977645 | 0.8023238 | 0.0490587 |
| MXRA8 | ENSG00000162576.16_2 | chr1:1288069-1297157 | 0.8026153 | 0.0414352 |
| DIAPH2 | ENSG00000147202.17_3 | chrX:95939662-96859996 | 0.8027297 | 0.0481176 |
| DKK3 | ENSG00000050165.17_2 | chr11:11984653-12031316 | 0.8040134 | 0.0166874 |
| PLPP7 | ENSG00000160539.4_2 | chr9:134165081-134184649 | 0.8070103 | 0.0397814 |
| VSIG10 | ENSG00000176834.13_3 | chr12:118501398-118573831 | 0.8070324 | 0.0147883 |
| WDR25 | ENSG00000176473.13_3 | chr14:100842755-100996640 | 0.8102412 | 0.0497626 |
| SFXN2 | ENSG00000156398.12_2 | chr10:104474295-104503249 | 0.8112266 | 0.0436299 |
| CADM2 | ENSG00000175161.13_3 | chr3:85008132-86123579 | 0.8117586 | 0.0058384 |
| DENR | ENSG00000139726.10_2 | chr12:123237321-123255611 | 0.8127315 | 0.0075447 |
| EMILIN3 | ENSG00000183798.4_2 | chr20:39988606-39995467 | 0.8131285 | 0.0025658 |
| SAT1 | ENSG00000130066.16_2 | chrX:23801290-23804343 | 0.8156705 | 0.0030396 |
| TMEM129 | ENSG00000168936.10_2 | chr4:1717679-1723085 | 0.8165839 | 0.0357883 |
| NCOA5 | ENSG00000124160.11_2 | chr20:44689624-44718591 | 0.816948 | 0.0218656 |
| TAF4B | ENSG00000141384.12_3 | chr18:23805900-23971649 | 0.8196568 | 0.0305958 |
| NFATC4 | ENSG00000100968.13_2 | chr14:24834879-24848810 | 0.821502 | 0.0311069 |
| S100B | ENSG00000160307.9_2 | chr21:48018875-48025121 | 0.8233767 | 0.0122193 |
| POLR2H | ENSG00000163882.9_2 | chr3:184079506-184086384 | 0.8262344 | 0.028745 |
| LMF1 | ENSG00000103227.18_3 | chr16:903634-1031318 | 0.8279473 | 0.0417975 |
| MLXIP | ENSG00000175727.9 | chr12:122516628-122631894 | 0.8280311 | 0.0250618 |
| MID1 | ENSG00000101871.14_3 | chrX:10413350-10851773 | 0.8284917 | 0.046098 |
| NOS1 | ENSG00000089250.18_3 | chr12:117645947-117889975 | 0.8286129 | 0.004469 |
| METTL22 | ENSG00000067365.14_3 | chr16:8715540-8743511 | 0.8290734 | 0.0068257 |
| SRF | ENSG00000112658.7_2 | chr6:43139037-43149245 | 0.8306528 | 0.022139 |
| PEX16 | ENSG00000121680.15_2 | chr11:45931220-45940363 | 0.8318244 | 0.0012978 |
| SIX5 | ENSG00000177045.7_2 | chr19:46268043-46272484 | 0.8319545 | 0.0217062 |
| SEMA6A | ENSG00000092421.16_2 | chr5:115779312-115910630 | 0.8323302 | 0.0232407 |
| SEMA6B | ENSG00000167680.15_2 | chr19:4542605-4559820 | 0.8360118 | 0.044608 |
| TBX3 | ENSG00000135111.15_4 | chr12:115108059-115121969 | 0.8369067 | 0.0286545 |
| CMTM3 | ENSG00000140931.19_3 | chr16:66637777-66647795 | 0.8391218 | 0.0030454 |
| CX3CL1 | ENSG00000006210.6_2 | chr16:57406370-57418960 | 0.839977 | 0.0130268 |
| IER3IP1 | ENSG00000134049.5_3 | chr18:44679205-44702745 | 0.8416676 | 0.0111585 |
| ETV5 | ENSG00000244405.7_3 | chr3:185764097-185828107 | 0.8430744 | 0.0209348 |
| ALG9 | ENSG00000086848.14_3 | chr11:111652919-111742305 | 0.8445512 | 0.0316643 |
| ZMYND10 | ENSG00000004838.13_2 | chr3:50378541-50384283 | 0.8446474 | 0.0271347 |
| ALG14 | ENSG00000172339.9_2 | chr1:95439963-95538501 | 0.8448063 | 0.0484718 |
| ARSE | ENSG00000157399.14_2 | chrX:2852673-2886351 | 0.8494884 | 0.0357613 |
| SLC7A11 | ENSG00000151012.13_2 | chr4:139085251-139163503 | 0.8500195 | 0.0155624 |
| ZBTB42 | ENSG00000179627.9_2 | chr14:105266933-105271049 | 0.853997 | 0.042856 |
| CCDC28A | ENSG00000024862.17_3 | chr6:139094646-139114456 | 0.8540457 | 0.0217784 |
| AKAP7 | ENSG00000118507.15_3 | chr6:131456806-131604675 | 0.8555378 | 0.0032681 |
| SNAPC1 | ENSG00000023608.4_3 | chr14:62229075-62263146 | 0.8558727 | 0.0496042 |
| EGLN3 | ENSG00000129521.13_3 | chr14:34393437-34931980 | 0.8562853 | 0.0313628 |
| ARHGAP29 | ENSG00000137962.12_2 | chr1:94614544-94740624 | 0.8602767 | 0.0357303 |
| STOML2 | ENSG00000165283.15_2 | chr9:35099773-35103192 | 0.8623024 | 0.0444955 |
| B9D2 | ENSG00000123810.7_2 | chr19:41860326-41870078 | 0.862856 | 0.0025211 |
| AK5 | ENSG00000154027.18_3 | chr1:77747736-78025654 | 0.8640915 | 0.047767 |
| NARF | ENSG00000141562.17_3 | chr17:80416056-80448413 | 0.8642955 | 0.0245191 |
| AHCYL1 | ENSG00000168710.17_2 | chr1:110527308-110566363 | 0.8657446 | 0.0234037 |
| PCOLCE | ENSG00000106333.12_2 | chr7:100199800-100205798 | 0.8663409 | 0.0304774 |
| HMOX1 | ENSG00000100292.16_2 | chr22:35776354-35790207 | 0.8685095 | 0.0341336 |
| SYMPK | ENSG00000125755.18_3 | chr19:46318668-46366548 | 0.8689492 | 0.0414298 |
| TRIM47 | ENSG00000132481.6_3 | chr17:73870242-73874656 | 0.8704927 | 0.018534 |
| ZMYND19 | ENSG00000165724.5_2 | chr9:140476531-140484942 | 0.8706221 | 0.0094699 |
| AGGF1 | ENSG00000164252.12_2 | chr5:76325076-76361059 | 0.8739965 | 0.0085216 |
| HDAC6 | ENSG00000094631.18_3 | chrX:48659784-48683392 | 0.8760633 | 0.0392684 |
| GMEB1 | ENSG00000162419.12_2 | chr1:28995244-29045865 | 0.8777809 | 0.0134855 |
| FAM204A | ENSG00000165669.13_2 | chr10:120057442-120101840 | 0.8804598 | 0.0371814 |
| IFT20 | ENSG00000109083.13_3 | chr17:26655351-26662515 | 0.8810979 | 0.016206 |
| CCDC71 | ENSG00000177352.9_2 | chr3:49199968-49203754 | 0.8826871 | 0.024342 |
| CLDN4 | ENSG00000189143.9_3 | chr7:73213872-73247023 | 0.890739 | 0.0488041 |
| TXNRD3 | ENSG00000197763.14_4 | chr3:126325895-126373998 | 0.8921462 | 0.0437159 |
| YPEL1 | ENSG00000100027.14_2 | chr22:22051833-22090123 | 0.8976422 | 0.022326 |
| MORN1 | ENSG00000116151.13_4 | chr1:2252692-2323146 | 0.9081944 | 0.0337996 |
| RNF175 | ENSG00000145428.14_3 | chr4:154631277-154681387 | 0.9097558 | 0.0131767 |
| KIAA0040 | ENSG00000235750.9_2 | chr1:175126123-175162135 | 0.9101844 | 0.0339572 |
| ACBD4 | ENSG00000181513.14_2 | chr17:43209967-43221548 | 0.9102199 | 0.0248649 |
| RCOR3 | ENSG00000117625.13_3 | chr1:211431719-211489727 | 0.9218826 | 0.0390127 |
| RBM6 | ENSG00000004534.14_3 | chr3:49977440-50137478 | 0.9270642 | 0.0474557 |
| ENY2 | ENSG00000120533.12_2 | chr8:110346553-110358182 | 0.9291883 | 0.0229105 |
| ST8SIA1 | ENSG00000111728.10_3 | chr12:22216707-22589975 | 0.9313841 | 0.0097897 |
| MAOB | ENSG00000069535.13_2 | chrX:43625858-43741693 | 0.9623278 | 0.0447987 |
