# Supplementary material for: Corticosterone-mediated regulation and functions of miR-218-5p in rat brain
Source: Sci Rep. 2022 Jan 7;12:194. doi: 10.1038/s41598-021-03863-y (PMC8742130; doi:10.1038/s41598-021-03863-y)
Supplement: Supplementary file 4 — Supplementary Table S3. [file 41598_2021_3863_MOESM4_ESM.docx]

**Table S3. All upregulated genes in IP RNA-seq result**

| **Gene symbol** | **Ensemble ID** | **Locus** | **Fold change** | **P value** |
| --- | --- | --- | --- | --- |
| **Upregulated genes** | | | | |
| ZNF3 | ENSG00000166526.16_3 | chr7:99661656-99680171 | 1.1068009 | 0.0086709 |
| RAB1B | ENSG00000174903.15_3 | chr11:66036004-66044963 | 1.1100756 | 0.0339554 |
| PCNX1 | ENSG00000100731.15_3 | chr14:71374122-71582099 | 1.1714002 | 0.0219741 |
| YARS | ENSG00000134684.10_2 | chr1:33240838-33283754 | 1.1735829 | 0.0450786 |
| PSMF1 | ENSG00000125818.17_3 | chr20:1093906-1170059 | 1.1859026 | 0.0297322 |
| VAPA | ENSG00000101558.13_2 | chr18:9913999-9960018 | 1.1975613 | 0.0357301 |
| SBF1 | ENSG00000100241.20_3 | chr22:50883429-50913453 | 1.2007565 | 0.0341363 |
| FOXK1 | ENSG00000164916.10_3 | chr7:4721940-4811074 | 1.2019066 | 0.0071526 |
| FAM234A | ENSG00000167930.15_3 | chr16:284545-318970 | 1.2025357 | 0.0472553 |
| RAD21 | ENSG00000164754.14_3 | chr8:117858174-117887105 | 1.2045857 | 0.0105873 |
| ORC4 | ENSG00000115947.13_3 | chr2:148687966-148779173 | 1.2146802 | 0.0322767 |
| BAG5 | ENSG00000166170.9_2 | chr14:104022881-104029168 | 1.2153379 | 0.0296926 |
| VPS35 | ENSG00000069329.16_3 | chr16:46690044-46723430 | 1.2173448 | 0.0499432 |
| UBR5 | ENSG00000104517.12_3 | chr8:103265240-103425069 | 1.2212073 | 0.0463649 |
| NCOA4 | ENSG00000266412.5_2 | chr10:51565108-51590734 | 1.226103 | 0.0325883 |
| TM7SF3 | ENSG00000064115.10_3 | chr12:27126128-27167367 | 1.2309066 | 0.0246754 |
| USP54 | ENSG00000166348.18_3 | chr10:75257296-75385711 | 1.236531 | 0.0313664 |
| DHX57 | ENSG00000163214.20_3 | chr2:39024871-39103075 | 1.2417207 | 0.0472286 |
| HDAC6 | ENSG00000094631.18_3 | chrX:48659784-48683392 | 1.2506261 | 0.0427818 |
| NBEAL1 | ENSG00000144426.18_3 | chr2:203879602-204091101 | 1.2509667 | 0.0496586 |
| KMT2E | ENSG00000005483.20_3 | chr7:104654626-104754808 | 1.2511408 | 0.0445262 |
| NANS | ENSG00000095380.10_2 | chr9:100819021-100845357 | 1.2533325 | 0.0001271 |
| ATP6V0A2 | ENSG00000185344.13_2 | chr12:124196865-124246302 | 1.25705 | 0.0043479 |
| SRCAP | ENSG00000080603.16_3 | chr16:30709530-30752730 | 1.258297 | 0.0034087 |
| TAOK1 | ENSG00000160551.11_3 | chr17:27717482-27878922 | 1.2637131 | 0.0423876 |
| BAP1 | ENSG00000163930.9_2 | chr3:52435029-52444366 | 1.2677558 | 0.0078303 |
| SMG1 | ENSG00000157106.12 | chr16:18816175-18937776 | 1.2697415 | 0.0020106 |
| PNMA2 | ENSG00000240694.8_2 | chr8:26362202-26371608 | 1.2700858 | 0.0176548 |
| OGFOD3 | ENSG00000181396.12_3 | chr17:80347099-80376513 | 1.2706435 | 0.045896 |
| PTEN | ENSG00000171862.10_3 | chr10:89622870-89731687 | 1.286269 | 0.0258182 |
| NARS | ENSG00000134440.11_3 | chr18:55267888-55289445 | 1.2883706 | 0.0199997 |
| TMCO6 | ENSG00000113119.12_2 | chr5:140019012-140024993 | 1.2887678 | 0.0080482 |
| ZMYM3 | ENSG00000147130.14_2 | chrX:70459474-70474996 | 1.2910579 | 0.0213642 |
| NEO1 | ENSG00000067141.16_2 | chr15:73344051-73597547 | 1.2926693 | 0.0322275 |
| KIAA0319L | ENSG00000142687.17_3 | chr1:35899091-36023551 | 1.2973204 | 0.0136898 |
| KCTD17 | ENSG00000100379.17_3 | chr22:37447776-37459430 | 1.3002357 | 0.0205666 |
| UBN1 | ENSG00000118900.14_3 | chr16:4896666-4932361 | 1.3031201 | 0.0205077 |
| IK | ENSG00000113141.17_3 | chr5:140026643-140042064 | 1.3188302 | 0.0293863 |
| KMT2C | ENSG00000055609.17_3 | chr7:151832007-152133090 | 1.3235499 | 0.0069977 |
| PER1 | ENSG00000179094.15_3 | chr17:8043790-8059824 | 1.3287934 | 0.0230163 |
| FARP1 | ENSG00000152767.16_3 | chr13:98794816-99107430 | 1.3307073 | 0.0447089 |
| ANKRD36C | ENSG00000174501.14_1 | chr2:96502667-96657579 | 1.3314326 | 0.0466308 |
| MYO19 | ENSG00000278259.4_2 | chr17:34851477-34899284 | 1.3330259 | 0.0331273 |
| ZNF646 | ENSG00000167395.10_3 | chr16:31085743-31095517 | 1.3336878 | 0.0077099 |
| BICD1 | ENSG00000151746.13_3 | chr12:32259769-32536567 | 1.3382338 | 0.0372834 |
| C16orf62 | ENSG00000103544.14_3 | chr16:19566562-19718115 | 1.3399062 | 0.0421928 |
| HERC3 | ENSG00000138641.15_3 | chr4:89442199-89629693 | 1.3404288 | 0.045583 |
| TACC3 | ENSG00000013810.18_3 | chr4:1723217-1746903 | 1.3415817 | 0.0345063 |
| ARHGAP28 | ENSG00000088756.12_2 | chr18:6729717-6915715 | 1.3450634 | 0.0343978 |
| KCND1 | ENSG00000102057.9_3 | chrX:48818639-48827976 | 1.3475722 | 0.0245944 |
| MTSS1L | ENSG00000132613.14_2 | chr16:70695107-70719969 | 1.3479535 | 0.009258 |
| SMARCA4 | ENSG00000127616.17_3 | chr19:11071677-11176071 | 1.3489761 | 0.0489786 |
| SYN2 | ENSG00000157152.16_3 | chr3:12045876-12232900 | 1.3491005 | 0.0244788 |
| IDI1 | ENSG00000067064.10_3 | chr10:1085848-1095110 | 1.3509136 | 0.0113485 |
| PDE5A | ENSG00000138735.15_2 | chr4:120415550-120550146 | 1.3510137 | 0.0321542 |
| BLVRA | ENSG00000106605.10_3 | chr7:43798279-43846941 | 1.3552201 | 0.0348902 |
| HIST1H2BG | ENSG00000187990.4 | chr6:26216428-26216872 | 1.3613731 | 0.0417256 |
| LTBP3 | ENSG00000168056.15_3 | chr11:65306276-65326401 | 1.3624797 | 0.0231341 |
| SLC20A1 | ENSG00000144136.10_3 | chr2:113403434-113421404 | 1.3667034 | 0.0411537 |
| KLF13 | ENSG00000169926.10_3 | chr15:31619058-31727868 | 1.3671815 | 0.0182817 |
| ANXA2 | ENSG00000182718.16_3 | chr15:60639333-60695082 | 1.367715 | 0.008119 |
| RPTOR | ENSG00000141564.13_2 | chr17:78518619-78940171 | 1.3728486 | 0.0499453 |
| PBXIP1 | ENSG00000163346.16_3 | chr1:154916552-154928599 | 1.3739849 | 0.0497256 |
| TTC3 | ENSG00000182670.13_3 | chr21:38445526-38575413 | 1.3753223 | 0.0424309 |
| GRINA | ENSG00000178719.16_3 | chr8:145064226-145067583 | 1.3783309 | 0.0414794 |
| NDST1 | ENSG00000070614.14_3 | chr5:149865381-149937773 | 1.3787266 | 0.0007252 |
| MRC2 | ENSG00000011028.13_2 | chr17:60704762-60770958 | 1.3823084 | 0.0120287 |
| TEX2 | ENSG00000136478.7_3 | chr17:62224587-62340661 | 1.3854198 | 0.0288004 |
| KLC1 | ENSG00000126214.21_3 | chr14:104028233-104180586 | 1.3854523 | 0.0082373 |
| PHKB | ENSG00000102893.15_3 | chr16:47495034-47735434 | 1.3880959 | 0.0072281 |
| ERI3 | ENSG00000117419.14_3 | chr1:44686742-44820932 | 1.3930756 | 0.0023625 |
| CSK | ENSG00000103653.16_3 | chr15:75074398-75095539 | 1.3942495 | 0.0367422 |
| CDC42BPB | ENSG00000198752.10_2 | chr14:103398716-103523799 | 1.3949804 | 0.012146 |
| PLSCR1 | ENSG00000188313.12_3 | chr3:146232967-146262651 | 1.3963081 | 0.0213504 |
| PMEPA1 | ENSG00000124225.15_3 | chr20:56223448-56286592 | 1.3964064 | 0.0483033 |
| ATP2A2 | ENSG00000174437.16_2 | chr12:110718561-110788898 | 1.3980395 | 0.0065847 |
| SURF1 | ENSG00000148290.9_3 | chr9:136218610-136223552 | 1.4016956 | 0.0005169 |
| LAYN | ENSG00000204381.11_3 | chr11:111411005-111432470 | 1.4028771 | 0.0062339 |
| WDR18 | ENSG00000065268.10_3 | chr19:984271-998437 | 1.4039187 | 0.0308161 |
| ZSWIM8 | ENSG00000214655.10_3 | chr10:75545340-75561555 | 1.412186 | 0.0161542 |
| MFSD8 | ENSG00000164073.10_3 | chr4:128838887-128887189 | 1.4139601 | 0.0211448 |
| FAM122B | ENSG00000156504.16_2 | chrX:133903596-133931262 | 1.4172361 | 0.0274075 |
| HAX1 | ENSG00000143575.14_3 | chr1:154244987-154248351 | 1.4197808 | 0.0116436 |
| SGK494 | ENSG00000167524.14_3 | chr17:26934982-26941218 | 1.4213036 | 0.0198757 |
| PITPNM1 | ENSG00000110697.12_2 | chr11:67259239-67273734 | 1.4215731 | 0.0147557 |
| P3H1 | ENSG00000117385.15_2 | chr1:43212006-43232755 | 1.4219014 | 0.0149768 |
| SMIM8 | ENSG00000111850.10_3 | chr6:88032301-88109467 | 1.4238821 | 0.0485987 |
| ATP2B1 | ENSG00000070961.15_2 | chr12:89981826-90103077 | 1.4249689 | 0.0173067 |
| MKRN3 | ENSG00000179455.7_2 | chr15:23810825-23875222 | 1.4286105 | 0.0183821 |
| GNG11 | ENSG00000127920.5_2 | chr7:93551011-93557922 | 1.4294222 | 0.0098895 |
| RGPD6 | ENSG00000183054.11_2 | chr2:111271389-111334762 | 1.4294637 | 0.0280959 |
| CTDSP1 | ENSG00000144579.7_3 | chr2:219262979-219270664 | 1.4308812 | 0.0001768 |
| ELK4 | ENSG00000158711.13_2 | chr1:205566684-205601090 | 1.43139 | 0.0083065 |
| CLSTN2 | ENSG00000158258.16_3 | chr3:139654027-140296239 | 1.4359508 | 0.0251307 |
| METTL22 | ENSG00000067365.14_3 | chr16:8715540-8743511 | 1.4386295 | 0.0354875 |
| AHCTF1 | ENSG00000153207.14_3 | chr1:247002400-247095280 | 1.4398563 | 0.0309779 |
| ERCC2 | ENSG00000104884.14_3 | chr19:45853095-45874176 | 1.4405644 | 0.0465691 |
| SOGA1 | ENSG00000149639.14_4 | chr20:35405845-35492089 | 1.4419498 | 0.0373895 |
| TAOK2 | ENSG00000149930.17_2 | chr16:29984962-30003582 | 1.4437381 | 0.0127231 |
| PELO | ENSG00000152684.10_3 | chr5:52083774-52099880 | 1.4474186 | 0.0486476 |
| CACYBP | ENSG00000116161.17_2 | chr1:174968300-174980851 | 1.448836 | 0.0167744 |
| CCNG2 | ENSG00000138764.14_3 | chr4:78078304-78354542 | 1.4500014 | 0.0237524 |
| MAGI3 | ENSG00000081026.18_3 | chr1:113933371-114228545 | 1.4507323 | 0.0378433 |
| KITLG | ENSG00000049130.14_2 | chr12:88886570-88974628 | 1.4524892 | 0.0420569 |
| PTPRN2 | ENSG00000155093.17_3 | chr7:157331750-158380480 | 1.4549479 | 0.0040651 |
| ZNF45 | ENSG00000124459.11_2 | chr19:44416781-44439430 | 1.4550586 | 0.0044676 |
| NCS1 | ENSG00000107130.9_2 | chr9:132934857-132999583 | 1.4558123 | 0.0340694 |
| CAPN5 | ENSG00000149260.15_3 | chr11:76777979-76837201 | 1.4600071 | 0.0461709 |
| ZNF438 | ENSG00000183621.15_3 | chr10:31109136-31320866 | 1.4616092 | 0.0271388 |
| DTWD1 | ENSG00000104047.14_2 | chr15:49913177-49948429 | 1.4631216 | 0.0269269 |
| TAGLN2 | ENSG00000158710.14_2 | chr1:159887897-159895522 | 1.4699942 | 0.040756 |
| MGAT4B | ENSG00000161013.16_3 | chr5:179224597-179233952 | 1.4710426 | 0.0079011 |
| IGSF8 | ENSG00000162729.13_3 | chr1:160061130-160068733 | 1.4715109 | 0.0257362 |
| KLLN | ENSG00000227268.3_2 | chr10:89618918-89623194 | 1.4735556 | 0.0368792 |
| RNF168 | ENSG00000163961.4_2 | chr3:196195654-196230639 | 1.477571 | 0.0353777 |
| NKRF | ENSG00000186416.13_3 | chrX:118722300-118739858 | 1.4787928 | 0.0331429 |
| LHFPL2 | ENSG00000145685.13_3 | chr5:77781038-78065844 | 1.4816132 | 0.0011024 |
| PANK2 | ENSG00000125779.21_2 | chr20:3869486-3910529 | 1.4831208 | 0.0108409 |
| KDM7A | ENSG00000006459.10_2 | chr7:139784546-139876835 | 1.4846078 | 0.0390777 |
| WDR34 | ENSG00000119333.11_2 | chr9:131395940-131419066 | 1.485663 | 0.0401103 |
| PCDHB16 | ENSG00000272674.3_3 | chr5:140560980-140565974 | 1.4871572 | 0.0446486 |
| SCARF2 | ENSG00000244486.8_3 | chr22:20779105-20792113 | 1.4875805 | 0.0033703 |
| KCNN3 | ENSG00000143603.18_3 | chr1:154669931-154842756 | 1.4894239 | 0.0106287 |
| RBM15B | ENSG00000259956.1_2 | chr3:51428699-51435339 | 1.4920436 | 0.0472616 |
| CAPN8 | ENSG00000203697.11_3 | chr1:223711349-223853436 | 1.4939287 | 0.0041994 |
| SYNM | ENSG00000182253.14_2 | chr15:99638420-99675798 | 1.4949466 | 0.008072 |
| SYNJ2BP | ENSG00000213463.4_3 | chr14:70833213-70883778 | 1.4965073 | 0.0438377 |
| ANKRD11 | ENSG00000167522.14_3 | chr16:89334035-89556969 | 1.4990089 | 0.0458269 |
| PPARGC1A | ENSG00000109819.8_2 | chr4:23756664-23905712 | 1.499371 | 0.0098949 |
| TMED2 | ENSG00000086598.10_2 | chr12:124069078-124083124 | 1.4994163 | 0.0388302 |
| HMG20B | ENSG00000064961.18_3 | chr19:3572775-3579086 | 1.5004641 | 0.0467742 |
| FCGBP | ENSG00000275395.5_3 | chr19:40353963-40440533 | 1.5007513 | 0.0415515 |
| CFAP44 | ENSG00000206530.10_3 | chr3:113005794-113160457 | 1.5054391 | 0.0067675 |
| ABI1 | ENSG00000136754.17_4 | chr10:27035522-27150016 | 1.5059012 | 0.046329 |
| GIGYF2 | ENSG00000204120.14_4 | chr2:233562009-233725285 | 1.5064577 | 0.0212261 |
| NUDT1 | ENSG00000106268.15_3 | chr7:2281857-2290781 | 1.5108492 | 0.0480687 |
| SYT16 | ENSG00000139973.16_3 | chr14:62278692-62579268 | 1.5153127 | 0.0352325 |
| MAP4K1 | ENSG00000104814.12_3 | chr19:39078281-39109522 | 1.5191203 | 0.0018996 |
| S100A10 | ENSG00000197747.8_2 | chr1:151955391-151966866 | 1.5210996 | 0.0223346 |
| MAPK4 | ENSG00000141639.11_3 | chr18:48086448-48258194 | 1.5212245 | 0.0262771 |
| APOOL | ENSG00000155008.13_2 | chrX:84258832-84348322 | 1.5227132 | 0.0169373 |
| LRRC29 | ENSG00000125122.15_3 | chr16:67241042-67260951 | 1.5231001 | 0.0044748 |
| LINGO1 | ENSG00000169783.12_3 | chr15:77905369-78113242 | 1.5232078 | 0.0172684 |
| HAUS5 | ENSG00000249115.8_3 | chr19:36103646-36116251 | 1.5238175 | 0.0227767 |
| TMEM218 | ENSG00000150433.9_2 | chr11:124966441-124981659 | 1.524115 | 0.0379798 |
| PFKP | ENSG00000067057.16_3 | chr10:3108525-3179904 | 1.5250646 | 0.0341426 |
| PLEKHJ1 | ENSG00000104886.11_3 | chr19:2230083-2237703 | 1.5273178 | 0.0360463 |
| TMEM127 | ENSG00000135956.8_2 | chr2:96914254-96931732 | 1.535601 | 0.0026805 |
| RAPGEF1 | ENSG00000107263.18_2 | chr9:134452157-134615461 | 1.5361971 | 0.0125429 |
| ACAP3 | ENSG00000131584.18_2 | chr1:1227756-1244989 | 1.536324 | 0.0479642 |
| EPB41L4A | ENSG00000129595.12_3 | chr5:111478138-111755013 | 1.5373233 | 0.0172084 |
| ARF3 | ENSG00000134287.9_3 | chr12:49329506-49351334 | 1.5391069 | 0.0321135 |
| NYAP1 | ENSG00000166924.8_2 | chr7:100081550-100092422 | 1.5447069 | 0.0034924 |
| TMEM245 | ENSG00000106771.12_2 | chr9:111777432-111882225 | 1.5448642 | 0.01942 |
| ZNF654 | ENSG00000175105.6_3 | chr3:88108424-88193815 | 1.5475508 | 0.0182656 |
| ANKRD9 | ENSG00000156381.8_3 | chr14:102968097-102976136 | 1.5476231 | 0.0158228 |
| CENPH | ENSG00000153044.9_2 | chr5:68485375-68506184 | 1.5497761 | 0.0043058 |
| PPP3CC | ENSG00000120910.14_3 | chr8:22298332-22398655 | 1.5534864 | 0.012319 |
| PDE4B | ENSG00000184588.17_3 | chr1:66258197-66840262 | 1.5540817 | 0.0435212 |
| CHKA | ENSG00000110721.11_2 | chr11:67820326-67888911 | 1.5551674 | 0.0204192 |
| NOTCH3 | ENSG00000074181.8_2 | chr19:15269849-15311792 | 1.5575991 | 0.0200691 |
| RBKS | ENSG00000171174.13_1 | chr2:28004231-28113965 | 1.5597871 | 0.0197589 |
| ZMYND11 | ENSG00000015171.19_3 | chr10:180405-300577 | 1.5612782 | 0.0064561 |
| ARMC8 | ENSG00000114098.17_2 | chr3:137906090-138017231 | 1.5625402 | 0.0418928 |
| ENTPD7 | ENSG00000198018.6_3 | chr10:101419263-101465997 | 1.5631955 | 0.0100029 |
| FAM126B | ENSG00000155744.9_2 | chr2:201838441-201936394 | 1.5656016 | 0.0212173 |
| CTDSPL | ENSG00000144677.14_2 | chr3:37903451-38025960 | 1.5691463 | 0.0112037 |
| SMIM20 | ENSG00000250317.8_2 | chr4:25863452-25931496 | 1.5706698 | 0.0187655 |
| REV3L | ENSG00000009413.15_3 | chr6:111620231-111804918 | 1.5710836 | 0.007443 |
| AKIP1 | ENSG00000166452.11_3 | chr11:8932686-8941631 | 1.5711677 | 0.0351448 |
| PKP2 | ENSG00000057294.14_3 | chr12:32943679-33049774 | 1.5714528 | 0.0144714 |
| KLHL2 | ENSG00000109466.13_3 | chr4:166128770-166244308 | 1.5715398 | 0.024875 |
| SLC2A4RG | ENSG00000125520.13_3 | chr20:62371214-62374858 | 1.575006 | 0.0320051 |
| ANXA6 | ENSG00000197043.13_2 | chr5:150480273-150537443 | 1.5760337 | 0.0209278 |
| PLEKHB1 | ENSG00000021300.13_3 | chr11:73357223-73373864 | 1.5761598 | 0.0436651 |
| SPOUT1 | ENSG00000198917.12_3 | chr9:131581930-131592100 | 1.5763333 | 0.0366043 |
| ZNF274 | ENSG00000171606.17_2 | chr19:58694396-58724928 | 1.5792679 | 0.024114 |
| MYADM | ENSG00000179820.15_3 | chr19:54369477-54379691 | 1.5806287 | 0.0349985 |
| GLIS3 | ENSG00000107249.21_3 | chr9:3824127-4348392 | 1.5811938 | 0.0336327 |
| CADM3 | ENSG00000162706.12_2 | chr1:159141399-159173103 | 1.5848847 | 0.0336039 |
| PLEKHA8 | ENSG00000106086.18_3 | chr7:30067020-30170099 | 1.5864409 | 0.0275748 |
| DOK6 | ENSG00000206052.10_3 | chr18:67068291-67516323 | 1.593179 | 0.0281296 |
| MAPK3 | ENSG00000102882.11_3 | chr16:30125426-30134827 | 1.5933762 | 0.0114097 |
| ITPR1 | ENSG00000150995.18_3 | chr3:4535032-4889524 | 1.5940659 | 0.0063938 |
| TSC22D2 | ENSG00000196428.12_2 | chr3:150126122-150184218 | 1.5946995 | 0.0409447 |
| ZNF157 | ENSG00000147117.7_2 | chrX:47229982-47273704 | 1.5969862 | 0.010855 |
| ATXN7 | ENSG00000163635.17_3 | chr3:63850233-63989138 | 1.5982838 | 0.0264733 |
| RHOU | ENSG00000116574.5_2 | chr1:228870824-228882416 | 1.5998891 | 0.0082501 |
| RALY | ENSG00000125970.11_4 | chr20:32581452-32696114 | 1.6011234 | 0.0359493 |
| TRAF3IP2 | ENSG00000056972.18_3 | chr6:111877657-111927481 | 1.6036119 | 0.0237098 |
| ATP5F1 | ENSG00000116459.10_2 | chr1:111991486-112005395 | 1.6059853 | 0.0075221 |
| FHL1 | ENSG00000022267.16_3 | chrX:135228861-135293518 | 1.6075477 | 0.0324133 |
| RAB12 | ENSG00000206418.3_2 | chr18:8609443-8639379 | 1.6091224 | 0.0376057 |
| PRAME | ENSG00000185686.17_4 | chr22:22890123-22901768 | 1.6111298 | 0.0451238 |
| PA2G4 | ENSG00000170515.13_3 | chr12:56498103-56507691 | 1.6116847 | 0.0391441 |
| NASP | ENSG00000132780.16_3 | chr1:46049518-46084576 | 1.6126115 | 0.0441927 |
| PSD | ENSG00000059915.16_3 | chr10:104162374-104181296 | 1.6127195 | 0.0406384 |
| ABCF1 | ENSG00000204574.12_3 | chr6:30539153-30564956 | 1.6148859 | 0.046326 |
| PQLC2L | ENSG00000174899.10_3 | chr3:157261035-157395538 | 1.6149105 | 0.0470744 |
| DYRK2 | ENSG00000127334.10_3 | chr12:68042118-68059186 | 1.6149628 | 0.038197 |
| AKAP6 | ENSG00000151320.10_3 | chr14:32798479-33306887 | 1.6177283 | 0.0232965 |
| BCL11A | ENSG00000119866.20_2 | chr2:60678302-60780702 | 1.625245 | 0.0098463 |
| CALU | ENSG00000128595.16_3 | chr7:128379346-128411861 | 1.6254245 | 0.0339946 |
| VPS4A | ENSG00000132612.15_3 | chr16:69345259-69360842 | 1.627095 | 0.016077 |
| APCDD1 | ENSG00000154856.12_4 | chr18:10454625-10489945 | 1.6295476 | 0.0364716 |
| MALT1 | ENSG00000172175.12_3 | chr18:56338618-56421709 | 1.6301404 | 0.010405 |
| HELZ2 | ENSG00000130589.16_2 | chr20:62189439-62205592 | 1.6315238 | 0.0485562 |
| ARL10 | ENSG00000175414.6_3 | chr5:175792471-175828866 | 1.6319847 | 0.0337983 |
| ASTN1 | ENSG00000152092.15_3 | chr1:176826438-177134109 | 1.6334944 | 0.0012318 |
| CCAR2 | ENSG00000158941.16_3 | chr8:22462145-22479027 | 1.6342707 | 0.0446925 |
| TSC1 | ENSG00000165699.13_2 | chr9:135766735-135820020 | 1.6394898 | 0.0460528 |
| SGPP1 | ENSG00000126821.7_2 | chr14:64150932-64194757 | 1.6425557 | 0.0372999 |
| ENTPD4 | ENSG00000197217.12_3 | chr8:23243296-23315208 | 1.6428477 | 0.0139199 |
| KCTD2 | ENSG00000180901.10_2 | chr17:73028670-73061984 | 1.6434797 | 0.0179247 |
| CEBPG | ENSG00000153879.8_2 | chr19:33864236-33873592 | 1.6440255 | 0.0269564 |
| SLC38A2 | ENSG00000134294.13_2 | chr12:46751972-46766650 | 1.6504653 | 0.0381842 |
| N4BP1 | ENSG00000102921.7_3 | chr16:48572637-48654059 | 1.6512023 | 0.0227859 |
| SAV1 | ENSG00000151748.14_3 | chr14:51098776-51135049 | 1.6530559 | 0.0451154 |
| POMP | ENSG00000132963.7_2 | chr13:29233241-29253062 | 1.6554838 | 0.0261498 |
| RAP1GAP | ENSG00000076864.19_3 | chr1:21922708-21995856 | 1.6571357 | 0.0071935 |
| TUBB6 | ENSG00000176014.12_3 | chr18:12307668-12344319 | 1.6581653 | 0.0100381 |
| TFEB | ENSG00000112561.17_3 | chr6:41651716-41703997 | 1.6612526 | 0.036693 |
| ACAD9 | ENSG00000177646.18_3 | chr3:128598439-128634910 | 1.6623672 | 0.0223435 |
| SMC5 | ENSG00000198887.8_3 | chr9:72873878-72969804 | 1.6627025 | 0.0322332 |
| FTSJ1 | ENSG00000068438.14_2 | chrX:48334409-48344752 | 1.6640733 | 0.0056783 |
| BCL2 | ENSG00000171791.12_3 | chr18:60790579-60987361 | 1.6659597 | 0.0381262 |
| LRR1 | ENSG00000165501.16_3 | chr14:50065415-50081390 | 1.6676587 | 0.0209357 |
| OSMR | ENSG00000145623.12_3 | chr5:38845960-38945698 | 1.668043 | 0.0169901 |
| CCDC186 | ENSG00000165813.18_3 | chr10:115880621-115933979 | 1.6717093 | 0.0043836 |
| CC2D2A | ENSG00000048342.15_3 | chr4:15471489-15603180 | 1.673687 | 0.035341 |
| ZNF707 | ENSG00000181135.15_4 | chr8:144766622-144796068 | 1.6757038 | 0.0485851 |
| DENND6A | ENSG00000174839.12_2 | chr3:57611184-57678816 | 1.6764615 | 0.0344035 |
| CD2AP | ENSG00000198087.7_3 | chr6:47445525-47594999 | 1.6777791 | 0.0133312 |
| PCDHB8 | ENSG00000120322.3_3 | chr5:140557371-140560110 | 1.6825122 | 0.0384185 |
| ZCCHC3 | ENSG00000247315.3_2 | chr20:277609-280965 | 1.6862157 | 0.0488161 |
| GBE1 | ENSG00000114480.12_3 | chr3:81538850-81811312 | 1.6884021 | 0.0291444 |
| OGFR | ENSG00000060491.16_3 | chr20:61436187-61445352 | 1.6906329 | 0.0325815 |
| MEIS3 | ENSG00000105419.17_3 | chr19:47906381-47922780 | 1.6911784 | 0.0267327 |
| RASSF1 | ENSG00000068028.17_2 | chr3:50367213-50378411 | 1.6926723 | 0.02519 |
| SCAMP3 | ENSG00000116521.10_2 | chr1:155225770-155232221 | 1.6936608 | 0.0458221 |
| MRPL53 | ENSG00000204822.6_3 | chr2:74699085-74700449 | 1.6942934 | 0.0239792 |
| GMPR | ENSG00000137198.9_2 | chr6:16238811-16295780 | 1.7006416 | 0.0173069 |
| CDC27 | ENSG00000004897.11_3 | chr17:45195069-45266788 | 1.7015092 | 0.047149 |
| ZFP92 | ENSG00000189420.8_2 | chrX:152683780-152691939 | 1.7051641 | 0.0349426 |
| FAM174B | ENSG00000185442.12_3 | chr15:93160673-93353114 | 1.7061193 | 0.0081219 |
| ANKRD12 | ENSG00000101745.16_3 | chr18:9136226-9285983 | 1.7170253 | 0.0077761 |
| BAG4 | ENSG00000156735.10_2 | chr8:38034051-38070819 | 1.722866 | 0.0475797 |
| UPP1 | ENSG00000183696.13_2 | chr7:48128225-48148330 | 1.7234233 | 0.0160509 |
| ESYT1 | ENSG00000139641.12_3 | chr12:56512034-56538455 | 1.7255519 | 0.049703 |
| UNC13D | ENSG00000092929.11_3 | chr17:73823306-73840798 | 1.7299606 | 0.0050701 |
| BOLA1 | ENSG00000178096.8_2 | chr1:149859440-149872351 | 1.7303217 | 0.0466813 |
| SPART | ENSG00000133104.12_3 | chr13:36875775-36944317 | 1.7308012 | 0.0068024 |
| CAPS2 | ENSG00000180881.19_4 | chr12:75669759-75784708 | 1.7327978 | 0.0088287 |
| APOL4 | ENSG00000100336.17_3 | chr22:36585172-36600886 | 1.7331294 | 0.0227054 |
| PIP5K1C | ENSG00000186111.9_3 | chr19:3630181-3700477 | 1.7343407 | 0.0335837 |
| TMEM63A | ENSG00000196187.11_3 | chr1:226033237-226070069 | 1.7356045 | 0.0017925 |
| CCDC14 | ENSG00000175455.14_4 | chr3:123616152-123680255 | 1.7382245 | 0.0018618 |
| RNF122 | ENSG00000133874.1_2 | chr8:33405273-33424643 | 1.7439287 | 0.033635 |
| UBE2O | ENSG00000175931.12_4 | chr17:74385532-74449288 | 1.7444435 | 0.0351627 |
| PDCD10 | ENSG00000114209.14_3 | chr3:167401086-167452727 | 1.7445122 | 0.0047547 |
| CRAT | ENSG00000095321.16_3 | chr9:131857089-131873468 | 1.7446619 | 0.0384207 |
| EGFL8 | ENSG00000241404.6_3 | chr6:32132360-32136058 | 1.7455347 | 7.704E-06 |
| PRELID3A | ENSG00000141391.13_3 | chr18:12407895-12432237 | 1.7471766 | 0.0142353 |
| MFSD1 | ENSG00000118855.18_3 | chr3:158449987-158547508 | 1.7471813 | 0.0402442 |
| SRSF3 | ENSG00000112081.16_2 | chr6:36562130-36573377 | 1.754702 | 0.0050125 |
| RGPD5 | ENSG00000015568.12_2 | chr2:110550335-110615272 | 1.7562517 | 0.0176837 |
| PML | ENSG00000140464.19_4 | chr15:74287014-74340153 | 1.7639999 | 0.0499941 |
| VDAC2 | ENSG00000165637.13_2 | chr10:76969912-76991206 | 1.7672438 | 0.0071638 |
| F3 | ENSG00000117525.13_2 | chr1:94994781-95007356 | 1.7686883 | 0.0358804 |
| REEP2 | ENSG00000132563.15_3 | chr5:137774706-137782658 | 1.7690777 | 0.0394379 |
| NLK | ENSG00000087095.12_2 | chr17:26368763-26523407 | 1.7707576 | 0.0047668 |
| DRAP1 | ENSG00000175550.7_2 | chr11:65686728-65689032 | 1.7710503 | 0.0457008 |
| CNEP1R1 | ENSG00000205423.11_2 | chr16:50058321-50070999 | 1.7720803 | 0.0416673 |
| INTS14 | ENSG00000138614.14_2 | chr15:65871091-65903627 | 1.7734103 | 0.0424988 |
| OVCA2 | ENSG00000262664.2_3 | chr17:1945230-1946724 | 1.7735213 | 0.0155458 |
| COLEC11 | ENSG00000118004.17_2 | chr2:3642422-3692234 | 1.7744404 | 0.0436694 |
| MTHFD2 | ENSG00000065911.11_2 | chr2:74425689-74444692 | 1.7746136 | 0.0496888 |
| E2F3 | ENSG00000112242.14_3 | chr6:20402137-20493946 | 1.7768813 | 0.0177286 |
| THOP1 | ENSG00000172009.14_2 | chr19:2785458-2815805 | 1.780067 | 0.0161298 |
| LACTB | ENSG00000103642.11_3 | chr15:63413999-63434260 | 1.7806805 | 0.0281234 |
| DUSP8 | ENSG00000184545.10_2 | chr11:1575274-1593150 | 1.7883104 | 0.038999 |
| PCID2 | ENSG00000126226.21_3 | chr13:113831853-113863029 | 1.7912125 | 0.0428401 |
| BCR | ENSG00000186716.20_3 | chr22:23521891-23660224 | 1.7923055 | 0.0095196 |
| PGPEP1 | ENSG00000130517.13_3 | chr19:18451397-18480760 | 1.7929776 | 0.0329556 |
| PRPSAP1 | ENSG00000161542.16_3 | chr17:74305567-74380602 | 1.7931272 | 0.0209154 |
| WRAP53 | ENSG00000141499.16_3 | chr17:7589389-7606820 | 1.7979294 | 0.0262152 |
| SCAMP5 | ENSG00000198794.11_3 | chr15:75249560-75313837 | 1.7988603 | 0.0444955 |
| PSENEN | ENSG00000205155.7_3 | chr19:36236015-36238420 | 1.8006755 | 0.0493364 |
| SPATA13 | ENSG00000182957.15_3 | chr13:24553944-24881212 | 1.8013509 | 0.0437189 |
| KLHDC9 | ENSG00000162755.13_2 | chr1:161068151-161070136 | 1.805864 | 0.0088954 |
| EFTUD2 | ENSG00000108883.12_2 | chr17:42927311-42977030 | 1.806648 | 0.0398349 |
| KATNAL1 | ENSG00000102781.13_3 | chr13:30776767-30881621 | 1.811092 | 0.0324284 |
| RNH1 | ENSG00000023191.16_2 | chr11:494512-507300 | 1.8159516 | 0.03311 |
| MGAT4A | ENSG00000071073.12_2 | chr2:99235569-99347589 | 1.8185699 | 0.01384 |
| THOC1 | ENSG00000079134.11_3 | chr18:214520-268050 | 1.8186459 | 0.0124239 |
| ITGA6 | ENSG00000091409.14_3 | chr2:173292082-173371010 | 1.8224774 | 0.0078288 |
| PSMB6 | ENSG00000142507.9_2 | chr17:4699439-4701798 | 1.8237638 | 0.0394059 |
| CEP131 | ENSG00000141577.13_3 | chr17:79163393-79196799 | 1.8238085 | 0.0250578 |
| PLEKHM1 | ENSG00000225190.10_3 | chr17:43513266-43568115 | 1.8245558 | 0.0019522 |
| FLYWCH1 | ENSG00000059122.16_2 | chr16:2961938-3001209 | 1.824989 | 0.016961 |
| LSM14A | ENSG00000257103.8_3 | chr19:34663409-34720420 | 1.8255409 | 0.0418203 |
| RGS17 | ENSG00000091844.7_2 | chr6:153325594-153452384 | 1.8259327 | 0.0411994 |
| VHL | ENSG00000134086.7_2 | chr3:10182692-10193904 | 1.8265091 | 0.0445698 |
| RABL3 | ENSG00000144840.8_2 | chr3:120405528-120461840 | 1.8269276 | 0.0053324 |
| IGFBP4 | ENSG00000141753.6_2 | chr17:38599713-38613983 | 1.8288655 | 0.0023517 |
| CPSF2 | ENSG00000165934.12_2 | chr14:92588281-92638489 | 1.831946 | 0.0470392 |
| PACRG | ENSG00000112530.11_3 | chr6:163148164-163736524 | 1.8321908 | 0.0317563 |
| SBK1 | ENSG00000188322.4_3 | chr16:28303840-28335170 | 1.8323322 | 0.030785 |
| ACACB | ENSG00000076555.15_3 | chr12:109554400-109706031 | 1.8397435 | 0.034842 |
| PLXNA1 | ENSG00000114554.11_2 | chr3:126707437-126756235 | 1.8418069 | 0.0082665 |
| ZIM2 | ENSG00000269699.5_3 | chr19:57285920-57352097 | 1.845662 | 0.0141449 |
| FUT4 | ENSG00000196371.3_3 | chr11:94277006-94283064 | 1.848928 | 0.0417453 |
| BROX | ENSG00000162819.11_3 | chr1:222885895-222908538 | 1.8542939 | 0.0462587 |
| MKRN2 | ENSG00000075975.15_2 | chr3:12598513-12625212 | 1.8633716 | 0.0038532 |
| SLC19A1 | ENSG00000173638.18_4 | chr21:46913486-46964325 | 1.8663091 | 0.0395069 |
| TBXA2R | ENSG00000006638.11_2 | chr19:3594504-3606838 | 1.8719487 | 0.0345937 |
| KLHL15 | ENSG00000174010.9_2 | chrX:24001837-24045303 | 1.8746404 | 0.0313149 |
| GXYLT1 | ENSG00000151233.10_2 | chr12:42475647-42538681 | 1.8759008 | 0.0158511 |
| FRYL | ENSG00000075539.14_4 | chr4:48499378-48782339 | 1.8795608 | 0.0197791 |
| KCNK6 | ENSG00000099337.4_2 | chr19:38810484-38822716 | 1.8818117 | 0.047816 |
| FAM111A | ENSG00000166801.15_2 | chr11:58910221-58922512 | 1.8888621 | 0.0169222 |
| PKN1 | ENSG00000123143.12_3 | chr19:14543865-14582679 | 1.8889026 | 0.0212352 |
| ZIK1 | ENSG00000171649.11_3 | chr19:58089824-58105145 | 1.8892609 | 0.0399536 |
| NUBP2 | ENSG00000095906.16_3 | chr16:1832902-1839192 | 1.8894129 | 0.037814 |
| PPP2R3B | ENSG00000167393.17_3 | chrX:294698-347690 | 1.8895054 | 0.0281737 |
| SGPP2 | ENSG00000163082.9_2 | chr2:223289236-223425667 | 1.8895735 | 0.0272601 |
| PPP2R3C | ENSG00000092020.10_3 | chr14:35554673-35591723 | 1.8920436 | 0.0173932 |
| PTAR1 | ENSG00000188647.12_2 | chr9:72324438-72374875 | 1.8954881 | 0.0296262 |
| SLC27A2 | ENSG00000140284.10_2 | chr15:50474393-50528592 | 1.902318 | 0.0312981 |
| ZNF81 | ENSG00000197779.13_2 | chrX:47696301-47861960 | 1.9025889 | 0.0378385 |
| CCDC126 | ENSG00000169193.11_3 | chr7:23636998-23684327 | 1.9060497 | 0.0101932 |
| CHDH | ENSG00000016391.10_3 | chr3:53846362-53880417 | 1.9073605 | 0.0231591 |
| ZBTB43 | ENSG00000169155.9_3 | chr9:129567285-129600489 | 1.9106965 | 0.0422646 |
| GOLGA3 | ENSG00000090615.14_4 | chr12:133345495-133405444 | 1.9111553 | 0.0464972 |
| MAP1B | ENSG00000131711.14_2 | chr5:71403061-71505397 | 1.9114208 | 0.0474599 |
| LMNTD1 | ENSG00000152936.10_3 | chr12:25562241-25801513 | 1.9121105 | 2.549E-05 |
| MED20 | ENSG00000124641.15_3 | chr6:41873092-41888877 | 1.9128486 | 0.044142 |
| GGA3 | ENSG00000125447.16_2 | chr17:73232694-73258444 | 1.9129266 | 0.0008949 |
| EFR3A | ENSG00000132294.14_3 | chr8:132916335-133025889 | 1.9154489 | 0.0415137 |
| SNX1 | ENSG00000028528.14_3 | chr15:64386322-64438289 | 1.9157549 | 0.0193768 |
| INPP1 | ENSG00000151689.12_2 | chr2:191208196-191236391 | 1.9234888 | 0.0210128 |
| CA1 | ENSG00000133742.13_3 | chr8:86239837-86291243 | 1.9307388 | 0.02873 |
| CDKL3 | ENSG00000006837.11_3 | chr5:133622041-133706738 | 1.9412305 | 0.0120965 |
| KIF3B | ENSG00000101350.7_2 | chr20:30865467-30922814 | 1.9418198 | 0.0265397 |
| SLC25A33 | ENSG00000171612.6_3 | chr1:9599541-9645237 | 1.9418361 | 0.0102532 |
| EDA2R | ENSG00000131080.14_2 | chrX:65815479-65859140 | 1.9419458 | 0.0055346 |
| LBR | ENSG00000143815.14_3 | chr1:225589204-225616627 | 1.9444696 | 0.02974 |
| PIK3CB | ENSG00000051382.8_2 | chr3:138371541-138553780 | 1.9473577 | 0.0207403 |
| SCAPER | ENSG00000140386.12_3 | chr15:76640245-77197785 | 1.9499303 | 0.0235003 |
| KIF5C | ENSG00000168280.12 | chr2:149632819-149883273 | 1.9520417 | 0.0150787 |
| MROH6 | ENSG00000204839.8_4 | chr8:144648357-144655141 | 1.9529137 | 0.0426287 |
| MTIF3 | ENSG00000122033.14_2 | chr13:28009780-28024728 | 1.9531183 | 0.0417002 |
| IRAK4 | ENSG00000198001.13_2 | chr12:44152747-44183346 | 1.9573209 | 0.038137 |
| HACL1 | ENSG00000131373.14_2 | chr3:15602211-15643359 | 1.9575115 | 0.007387 |
| RCBTB2 | ENSG00000136161.12_3 | chr13:49063095-49107392 | 1.9585841 | 0.0417051 |
| MDM1 | ENSG00000111554.14_2 | chr12:68666223-68726161 | 1.9628767 | 0.023321 |
| IRS2 | ENSG00000185950.8_2 | chr13:110405045-110438915 | 1.9650294 | 0.0392489 |
| TLNRD1 | ENSG00000140406.3_3 | chr15:81293285-81298129 | 1.9674237 | 0.0101799 |
| PLXNB2 | ENSG00000196576.14_2 | chr22:50713408-50746056 | 1.9678204 | 0.0254169 |
| CYFIP2 | ENSG00000055163.19_3 | chr5:156693089-156822603 | 1.9708906 | 0.0222791 |
| PDRG1 | ENSG00000088356.5_2 | chr20:30532145-30539895 | 1.9722811 | 0.0296869 |
| YKT6 | ENSG00000106636.7_3 | chr7:44240567-44253893 | 1.977581 | 0.0149804 |
| FANK1 | ENSG00000203780.10_3 | chr10:127585108-127698161 | 1.9797683 | 0.0011097 |
| PGF | ENSG00000119630.13_2 | chr14:75408537-75422487 | 1.9880646 | 0.0478507 |
| GAS6 | ENSG00000183087.14_2 | chr13:114523522-114567040 | 1.9884158 | 0.0365376 |
| ARG2 | ENSG00000081181.7_2 | chr14:68086515-68118437 | 1.9979921 | 0.0394771 |
| RIC1 | ENSG00000107036.11_2 | chr9:5629025-5776557 | 2.0010346 | 0.0451138 |
| SAMD9 | ENSG00000205413.7_3 | chr7:92728826-92747336 | 2.0079724 | 0.0066384 |
| ANO10 | ENSG00000160746.12_3 | chr3:43396351-43733086 | 2.0110098 | 0.0087077 |
| TMEM151B | ENSG00000178233.17_3 | chr6:44238203-44275243 | 2.0124335 | 0.0381863 |
| TRIQK | ENSG00000205133.11_3 | chr8:93895758-94029901 | 2.0160817 | 0.0457636 |
| BMT2 | ENSG00000164603.11_3 | chr7:112459202-112579971 | 2.0173569 | 0.0396358 |
| AGPAT4 | ENSG00000026652.13_3 | chr6:161551011-161695093 | 2.0194428 | 0.0190627 |
| METTL12 | ENSG00000214756.7_2 | chr11:62432781-62435968 | 2.0216879 | 0.0361143 |
| SPECC1L | ENSG00000100014.19_4 | chr22:24666785-24813708 | 2.0223969 | 0.0384129 |
| C14orf79 | ENSG00000140104.13_3 | chr14:105452112-105476819 | 2.0227331 | 0.0136444 |
| VAMP2 | ENSG00000220205.8_3 | chr17:8062467-8066864 | 2.0257181 | 0.0296982 |
| MIDN | ENSG00000167470.12_3 | chr19:1248552-1259139 | 2.0318653 | 0.0129677 |
| SLC36A1 | ENSG00000123643.12_3 | chr5:150816607-150871942 | 2.0333222 | 0.0163171 |
| JAKMIP2 | ENSG00000176049.15_2 | chr5:146965002-147162411 | 2.0347442 | 0.0188399 |
| STARD10 | ENSG00000214530.8_3 | chr11:72465774-72505213 | 2.0412466 | 0.0190742 |
| MRPL28 | ENSG00000086504.15_2 | chr16:417384-420527 | 2.0412876 | 0.0303467 |
| AASDH | ENSG00000157426.13_2 | chr4:57204453-57253674 | 2.0445973 | 0.000537 |
| MAGED4B | ENSG00000187243.16_3 | chrX:51804923-51812344 | 2.044764 | 0.0030781 |
| FRMD8 | ENSG00000126391.13_2 | chr11:65154070-65180996 | 2.04497 | 0.0027032 |
| KMT5C | ENSG00000133247.13_3 | chr19:55851221-55859488 | 2.0452342 | 0.0129556 |
| TRO | ENSG00000067445.20_4 | chrX:54946895-54957864 | 2.049251 | 0.0460672 |
| KIF26B | ENSG00000162849.15_3 | chr1:245318287-245872733 | 2.0492858 | 0.0188366 |
| BMP8B | ENSG00000116985.11_3 | chr1:40222854-40254533 | 2.0516974 | 0.019278 |
| HSD3B1 | ENSG00000203857.9_2 | chr1:120049821-120057677 | 2.0523574 | 0.0393198 |
| CEP126 | ENSG00000110318.13_2 | chr11:101785746-101871789 | 2.0551161 | 0.0289273 |
| ADPGK | ENSG00000159322.17_2 | chr15:73043710-73078187 | 2.0571703 | 0.0184747 |
| SPATA18 | ENSG00000163071.10_2 | chr4:52917497-52963458 | 2.0651609 | 0.0163328 |
| RDH5 | ENSG00000135437.9_3 | chr12:56114151-56118489 | 2.0675612 | 0.0256055 |
| CENPL | ENSG00000120334.15_2 | chr1:173768688-173793858 | 2.0725083 | 0.0340755 |
| MPDU1 | ENSG00000129255.15_3 | chr17:7486847-7496107 | 2.0756215 | 0.0124705 |
| SSH2 | ENSG00000141298.17_3 | chr17:27952956-28257294 | 2.0775764 | 0.0376738 |
| ACVR2B | ENSG00000114739.13_2 | chr3:38495342-38534633 | 2.0948074 | 0.038902 |
| MCRS1 | ENSG00000187778.13_2 | chr12:49950327-49961936 | 2.0977376 | 0.023829 |
| CNPY4 | ENSG00000166997.7_3 | chr7:99717236-99723134 | 2.1064476 | 0.002595 |
| PPP1R18 | ENSG00000146112.11_2 | chr6:30644166-30655672 | 2.1083535 | 0.043193 |
| PDE7B | ENSG00000171408.13_3 | chr6:136172834-136516712 | 2.1117629 | 0.0091385 |
| FAM20C | ENSG00000177706.8 | chr7:192969-300711 | 2.1136347 | 0.0362801 |
| FOXO6 | ENSG00000204060.7_4 | chr1:41827594-41849262 | 2.1182645 | 0.0355229 |
| CLTA | ENSG00000122705.16_2 | chr9:36190853-36304778 | 2.1189818 | 0.0020358 |
| TMEM80 | ENSG00000177042.14_3 | chr11:695428-705028 | 2.1347258 | 0.002046 |
| ARNTL2 | ENSG00000029153.14_3 | chr12:27485787-27578222 | 2.1420543 | 0.0202906 |
| STX16 | ENSG00000124222.22_3 | chr20:57226309-57254582 | 2.1424785 | 0.0084253 |
| PIK3CD | ENSG00000171608.15_2 | chr1:9711790-9789172 | 2.143322 | 0.0128971 |
| PIM3 | ENSG00000198355.4_2 | chr22:50354161-50357728 | 2.1491508 | 0.0467677 |
| ZC3H18 | ENSG00000158545.15_4 | chr16:88636789-88698374 | 2.163249 | 0.0228899 |
| MAN2A2 | ENSG00000196547.14_3 | chr15:91445448-91465814 | 2.1648105 | 0.0277266 |
| ATXN1L | ENSG00000224470.7_3 | chr16:71879894-71919171 | 2.1655825 | 0.0402512 |
| OLFM2 | ENSG00000105088.8_2 | chr19:9964394-10047228 | 2.170035 | 0.0286293 |
| UVRAG | ENSG00000198382.8_3 | chr11:75526212-75854239 | 2.1746701 | 0.0476562 |
| RARB | ENSG00000077092.18_3 | chr3:25215823-25639423 | 2.1815774 | 0.0016502 |
| ARID3A | ENSG00000116017.6 | chr19:925781-975939 | 2.183074 | 0.002764 |
| ENGASE | ENSG00000167280.16_3 | chr17:77071021-77084681 | 2.1885173 | 0.0135598 |
| PEX11A | ENSG00000166821.8_3 | chr15:90220995-90234014 | 2.1962582 | 0.042688 |
| PTGER2 | ENSG00000125384.6_2 | chr14:52781023-52795324 | 2.1975382 | 0.0456745 |
| TGFB1 | ENSG00000105329.9_2 | chr19:41807492-41859816 | 2.1988127 | 0.0466987 |
| TEC | ENSG00000135605.12_2 | chr4:48137800-48271881 | 2.2026867 | 0.0398249 |
| GMIP | ENSG00000089639.10_2 | chr19:19740285-19754476 | 2.2047351 | 0.0414339 |
| KTI12 | ENSG00000198841.3_3 | chr1:52497775-52499488 | 2.2169572 | 0.0391032 |
| GABRP | ENSG00000094755.16_3 | chr5:170190354-170241051 | 2.2177813 | 0.0273896 |
| CIAO1 | ENSG00000144021.2_2 | chr2:96931870-96939087 | 2.2271006 | 0.0196238 |
| USE1 | ENSG00000053501.12_2 | chr19:17326155-17330638 | 2.2337045 | 0.0244329 |
| CALD1 | ENSG00000122786.19_3 | chr7:134429003-134655479 | 2.2352421 | 0.004066 |
| DYNC2LI1 | ENSG00000138036.18_2 | chr2:44001178-44037149 | 2.2386355 | 0.0297202 |
| KLHL28 | ENSG00000179454.13_2 | chr14:45393522-45511525 | 2.2494342 | 0.0389021 |
| SPICE1 | ENSG00000163611.11_2 | chr3:113161565-113234034 | 2.249464 | 0.0181351 |
| CROCC | ENSG00000058453.16_3 | chr1:17066768-17299474 | 2.2515031 | 0.0368606 |
| KCNJ11 | ENSG00000187486.5_2 | chr11:17407406-17410878 | 2.2565282 | 0.0442309 |
| ECHDC3 | ENSG00000134463.14_3 | chr10:11784365-11806069 | 2.258532 | 0.0381928 |
| AK7 | ENSG00000140057.8_2 | chr14:96858448-96955764 | 2.2600632 | 0.0167129 |
| CDKN1A | ENSG00000124762.13_2 | chr6:36644237-36655116 | 2.267838 | 0.0247124 |
| GUCA1A | ENSG00000048545.13_3 | chr6:42123144-42147794 | 2.2728276 | 0.0072125 |
| C9orf64 | ENSG00000165118.14_2 | chr9:86553226-86571901 | 2.27313 | 0.0457992 |
| NUBPL | ENSG00000151413.16_3 | chr14:31959162-32330430 | 2.2847008 | 0.0182462 |
| EXOSC9 | ENSG00000123737.12_3 | chr4:122722472-122738176 | 2.2892456 | 0.010617 |
| BNIP3 | ENSG00000176171.11_2 | chr10:133779959-133795517 | 2.299012 | 0.0130285 |
| SOGA3 | ENSG00000255330.9_3 | chr6:127759551-127840146 | 2.3068626 | 0.0300583 |
| HES7 | ENSG00000179111.8_3 | chr17:8023908-8027410 | 2.3131418 | 0.0054706 |
| ZC2HC1A | ENSG00000104427.11_2 | chr8:79578282-79632000 | 2.3144666 | 0.0350223 |
| C16orf52 | ENSG00000185716.11_2 | chr16:22018959-22098855 | 2.3160835 | 0.0021857 |
| AFAP1L1 | ENSG00000157510.13_2 | chr5:148651434-148721365 | 2.3264541 | 0.0180977 |
| RCAN1 | ENSG00000159200.17_2 | chr21:35885440-35987441 | 2.3270523 | 0.0072934 |
| MAPKAPK2 | ENSG00000162889.10_2 | chr1:206858289-206907628 | 2.3530393 | 0.0020412 |
| VPS9D1 | ENSG00000075399.13_4 | chr16:89773542-89787394 | 2.3549701 | 0.0199921 |
| ANKRD45 | ENSG00000183831.6_2 | chr1:173578700-173639001 | 2.3593226 | 0.0069019 |
| LMO4 | ENSG00000143013.12_2 | chr1:87794151-87814606 | 2.3617565 | 0.0345786 |
| ANKRD42 | ENSG00000137494.13_3 | chr11:82904781-82971736 | 2.367345 | 0.0296041 |
| ARPC5 | ENSG00000162704.15_3 | chr1:183589981-183604892 | 2.3697363 | 0.0071656 |
| AUH | ENSG00000148090.11_2 | chr9:93976097-94124195 | 2.3700304 | 0.0182758 |
| TCTE3 | ENSG00000184786.5_2 | chr6:170140210-170151683 | 2.3810332 | 0.0412719 |
| METRN | ENSG00000103260.8_4 | chr16:765115-769655 | 2.3811438 | 0.0147267 |
| STK35 | ENSG00000125834.12_2 | chr20:2082257-2157684 | 2.3834844 | 0.0393732 |
| ABHD6 | ENSG00000163686.14_3 | chr3:58223233-58281420 | 2.38498 | 0.0032952 |
| ZNF695 | ENSG00000197472.14_3 | chr1:247108849-247171395 | 2.3899524 | 0.0020978 |
| AP5B1 | ENSG00000254470.2_2 | chr11:65543364-65548273 | 2.3913179 | 0.0054588 |
| SLC35A1 | ENSG00000164414.17_3 | chr6:88180341-88222054 | 2.3928508 | 0.0103244 |
| C2CD4C | ENSG00000183186.7_2 | chr19:405438-409170 | 2.3976961 | 0.0214752 |
| AADAT | ENSG00000109576.13_2 | chr4:170981373-171012850 | 2.410961 | 0.0323987 |
| ZNF514 | ENSG00000144026.11_4 | chr2:95813075-95831158 | 2.4127842 | 0.0263885 |
| ZNF680 | ENSG00000173041.11_2 | chr7:63980262-64023484 | 2.4217611 | 0.0090093 |
| BLOC1S2 | ENSG00000196072.11_3 | chr10:102033037-102046469 | 2.4286756 | 0.0344946 |
| TMCC1 | ENSG00000172765.16_3 | chr3:129366635-129612419 | 2.4340111 | 0.0156276 |
| KPNA5 | ENSG00000196911.10_3 | chr6:117002350-117063029 | 2.439181 | 0.0299923 |
| NUDT17 | ENSG00000186364.11_2 | chr1:145586115-145589439 | 2.4420006 | 0.0234466 |
| DTX2 | ENSG00000091073.19_3 | chr7:76090993-76135312 | 2.4430808 | 0.0423155 |
| CDON | ENSG00000064309.14_2 | chr11:125825691-125933230 | 2.4510188 | 0.0115533 |
| SRRM3 | ENSG00000177679.15_3 | chr7:75831218-75916605 | 2.4559742 | 0.0413239 |
| RRM2B | ENSG00000048392.11_2 | chr8:103216730-103251346 | 2.4641355 | 0.0463366 |
| KDELR3 | ENSG00000100196.10_2 | chr22:38864067-38879452 | 2.4724369 | 0.0232534 |
| SLX4 | ENSG00000188827.10_2 | chr16:3631182-3661599 | 2.4761376 | 0.0414887 |
| KAT5 | ENSG00000172977.12_3 | chr11:65479467-65487075 | 2.4773622 | 0.0028062 |
| CDK11A | ENSG00000008128.22_3 | chr1:1634169-1655763 | 2.4855285 | 0.002648 |
| ZNF439 | ENSG00000171291.8_3 | chr19:11959541-11994565 | 2.4926376 | 0.0268365 |
| FAM27E1 | ENSG00000237198.2 | chr9:46385691-46387031 | 2.4943536 | 0.0062173 |
| PELP1 | ENSG00000141456.14_3 | chr17:4573069-4607632 | 2.4971326 | 0.0107568 |
| TBC1D22A | ENSG00000054611.13_2 | chr22:47158514-47571342 | 2.5010228 | 0.0359449 |
| BCAT2 | ENSG00000105552.14_3 | chr19:49298319-49314286 | 2.5158571 | 0.0083771 |
| RABAC1 | ENSG00000105404.10_2 | chr19:42460833-42463542 | 2.5165276 | 0.0315796 |
| DNAAF4 | ENSG00000256061.7_4 | chr15:55702723-55800432 | 2.5255155 | 0.0091281 |
| ZFP30 | ENSG00000120784.15_3 | chr19:38104650-38183238 | 2.5322358 | 0.0152108 |
| TLCD1 | ENSG00000160606.10_2 | chr17:27051366-27054953 | 2.5473779 | 0.0088628 |
| APAF1 | ENSG00000120868.13_3 | chr12:99038919-99129211 | 2.554373 | 0.0271007 |
| SEC61A2 | ENSG00000065665.20_3 | chr10:12171636-12211960 | 2.5777508 | 0.0202101 |
| PLXNA2 | ENSG00000076356.6_2 | chr1:208195587-208417665 | 2.5953632 | 0.0356925 |
| ZNF708 | ENSG00000182141.10_3 | chr19:21473962-21512227 | 2.6093516 | 0.0064619 |
| ETFDH | ENSG00000171503.11_4 | chr4:159593277-159630775 | 2.621538 | 0.0235775 |
| DTNBP1 | ENSG00000047579.19_2 | chr6:15523032-15663289 | 2.6231352 | 0.0325901 |
| ABCF3 | ENSG00000161204.11_2 | chr3:183903811-183911800 | 2.6331117 | 0.0247936 |
| RD3 | ENSG00000198570.5_2 | chr1:211649864-211666259 | 2.6510922 | 0.0322879 |
| CNTFR | ENSG00000122756.14_3 | chr9:34551430-34590138 | 2.6561584 | 0.0366103 |
| DCUN1D2 | ENSG00000150401.14_3 | chr13:114110134-114145267 | 2.6596187 | 0.0192038 |
| ZNF408 | ENSG00000175213.2_2 | chr11:46722368-46727462 | 2.6624589 | 0.0152317 |
| PEX14 | ENSG00000142655.12_2 | chr1:10532345-10690815 | 2.6806145 | 0.0444326 |
| HAUS7 | ENSG00000213397.10_4 | chrX:152713124-152760974 | 2.6837578 | 0.0287867 |
| SPRY2 | ENSG00000136158.11_3 | chr13:80910111-80915086 | 2.7077379 | 0.0151234 |
| ASF1A | ENSG00000111875.7_2 | chr6:119215384-119230332 | 2.7246009 | 0.01046 |
| DSTN | ENSG00000125868.15_2 | chr20:17550508-17590564 | 2.7457984 | 0.0422087 |
| AL162231.1 | ENSG00000187186.14_4 | chr9:34664160-34666109 | 2.7485328 | 0.0385304 |
| ZNF229 | ENSG00000278318.4_3 | chr19:44921685-44952766 | 2.782018 | 0.0123458 |
| CAPN12 | ENSG00000182472.8_3 | chr19:39220827-39260544 | 2.7844923 | 0.032496 |
| COQ7 | ENSG00000167186.10_3 | chr16:19078921-19091417 | 2.7982088 | 0.048597 |
| NEFL | ENSG00000277586.2_3 | chr8:24808468-24814624 | 2.8148477 | 0.0047071 |
| CLDN3 | ENSG00000165215.6_3 | chr7:73183327-73184600 | 2.8183852 | 0.0236509 |
| AC027796.3 | ENSG00000262304.2_4 | chr17:3488443-3539543 | 2.8394509 | 0.0145925 |
| ZNF329 | ENSG00000181894.14_2 | chr19:58637619-58666477 | 2.8662799 | 0.0356353 |
| SLC22A18 | ENSG00000110628.13_3 | chr11:2920951-2946476 | 2.8671695 | 0.0428674 |
| DNPEP | ENSG00000123992.18_2 | chr2:220238268-220264744 | 2.8852845 | 0.0043239 |
| PEG10 | ENSG00000242265.5_3 | chr7:94285637-94299007 | 2.9259308 | 0.0172003 |
| NET1 | ENSG00000173848.18_2 | chr10:5454514-5500426 | 2.9359825 | 0.0098272 |
| EIF2B3 | ENSG00000070785.16_3 | chr1:45316194-45452394 | 2.9718132 | 0.002439 |
| INSIG2 | ENSG00000125629.14_2 | chr2:118846028-118868573 | 2.9736449 | 0.0230991 |
| CRABP2 | ENSG00000143320.8_2 | chr1:156669398-156675608 | 2.9745254 | 0.0062075 |
| FAM124A | ENSG00000150510.16_3 | chr13:51796470-51858377 | 2.9799421 | 0.0163269 |
| ZNF92 | ENSG00000146757.13_2 | chr7:64838712-64866048 | 2.9803818 | 0.0469691 |
| PARPBP | ENSG00000185480.11_2 | chr12:102513963-102591298 | 2.9840834 | 0.0477598 |
| HABP4 | ENSG00000130956.13_2 | chr9:99212483-99253618 | 3.0398252 | 0.0212902 |
| ALG10B | ENSG00000175548.8_2 | chr12:38710380-38723530 | 3.0426594 | 0.0028828 |
| PLA2G16 | ENSG00000176485.11_3 | chr11:63340667-63384355 | 3.0438544 | 0.0222136 |
| ZNF433 | ENSG00000197647.11_3 | chr19:12125547-12146556 | 3.049583 | 0.0149181 |
| MOXD1 | ENSG00000079931.14_2 | chr6:132617194-132722684 | 3.0541212 | 0.0205254 |
| ATP13A2 | ENSG00000159363.17_3 | chr1:17312453-17338423 | 3.0610524 | 0.0164827 |
| OPRL1 | ENSG00000125510.15_2 | chr20:62711526-62731996 | 3.0648349 | 0.0276203 |
| ALG10 | ENSG00000139133.6_2 | chr12:34175216-34182629 | 3.0992014 | 0.0177937 |
| LSM10 | ENSG00000181817.5_2 | chr1:36856839-36863493 | 3.1005365 | 0.0110833 |
| KLF16 | ENSG00000129911.8_2 | chr19:1852398-1863567 | 3.1327074 | 0.0492787 |
| CPEB2 | ENSG00000137449.15_2 | chr4:15004298-15071777 | 3.1494193 | 0.0485707 |
| SNX10 | ENSG00000086300.15_2 | chr7:26331515-26413949 | 3.2139584 | 0.0196472 |
| SNAPC1 | ENSG00000023608.4_3 | chr14:62229075-62263146 | 3.2348084 | 0.0269608 |
| RAB36 | ENSG00000100228.12_2 | chr22:23487513-23506537 | 3.2687366 | 0.0068932 |
| GMPPB | ENSG00000173540.12_3 | chr3:49754277-49761384 | 3.2898078 | 0.002338 |
| SAMD10 | ENSG00000130590.13_3 | chr20:62605466-62611361 | 3.3666888 | 0.0138254 |
| GPATCH3 | ENSG00000198746.12_3 | chr1:27216979-27226957 | 3.3763432 | 0.0081224 |
| RELL2 | ENSG00000164620.8_2 | chr5:141016517-141020644 | 3.4950355 | 0.022839 |
| HES1 | ENSG00000114315.3_2 | chr3:193853934-193856521 | 3.5050307 | 0.0150349 |
| CCDC188 | ENSG00000234409.6_2 | chr22:20135950-20138588 | 3.5827173 | 0.0009169 |
| ZNF564 | ENSG00000249709.7_3 | chr19:12636184-12662356 | 3.6500361 | 0.0023232 |
| NIPA1 | ENSG00000170113.15_4 | chr15:23043277-23100005 | 3.6561212 | 0.0039284 |
| RRAGD | ENSG00000025039.14_2 | chr6:90074355-90121989 | 3.6583981 | 0.0490655 |
| PPP1R15A | ENSG00000087074.7_3 | chr19:49375649-49379314 | 3.7126537 | 0.0394323 |
| BNIP1 | ENSG00000113734.17_3 | chr5:172571445-172591390 | 3.7688076 | 0.0018831 |
| TUSC1 | ENSG00000198680.4_2 | chr9:25676387-25678438 | 3.9140432 | 0.0462853 |
| ENC1 | ENSG00000171617.13_3 | chr5:73923231-73937249 | 3.9799068 | 0.0243235 |
| EPOR | ENSG00000187266.13_3 | chr19:11487881-11495018 | 4.0292841 | 0.003443 |
| MYPOP | ENSG00000176182.5_2 | chr19:46393278-46405862 | 4.0619261 | 0.0496965 |
| SYVN1 | ENSG00000162298.18_3 | chr11:64889252-64902004 | 4.13482 | 0.0009663 |
| SESN2 | ENSG00000130766.4_2 | chr1:28586038-28609002 | 4.1859227 | 0.0328403 |
| METRNL | ENSG00000176845.12_2 | chr17:81037567-81052864 | 4.2145826 | 0.0396011 |
| KIFC2 | ENSG00000167702.11_2 | chr8:145691426-145699585 | 4.3495903 | 0.0179832 |
| RAB33A | ENSG00000134594.4_2 | chrX:129305623-129318844 | 4.3894855 | 0.0074479 |
| LCMT1 | ENSG00000205629.11_2 | chr16:25123050-25189552 | 4.4675085 | 0.0429618 |
| ZNF490 | ENSG00000188033.9_3 | chr19:12688775-12750912 | 5.0022176 | 0.0012001 |
| CENPQ | ENSG00000031691.6_2 | chr6:49431091-49460820 | 5.0039877 | 0.0006448 |
| FDX2 | ENSG00000267673.6_3 | chr19:10420721-10426691 | 5.9437033 | 0.0034942 |
| DDTL | ENSG00000099974.7_3 | chr22:24309089-24314721 | 6.4066963 | 0.0079743 |
| PDF | ENSG00000258429.1_3 | chr16:69362524-69364498 | 11.527763 | 0.0161186 |
